# ATR inhibition induces NF-κB-dependent immunogenicity in Merkel cell carcinoma independent of cGAS-STING

**DOI:** 10.1038/s41698-026-01688-4

**Published:** 2026-09-12

**Authors:** Rashmi Bhakuni, Jung Hyun Lee, Peter Goff, Daniel Jones, Long Nguyen, Rongxiang Lin, Evan W. Newell, Khalid A. Garman, Evan T. Hall, Oren Gilad, Isaac Brownell, Eric J. Brown, Paul Nghiem

**Affiliations:** 1 Department of Dermatology, University of Washington, Seattle, WA USA; 2 University of Washington, Seattle, WA USA; 3 Department of Radiation Oncology, University of Washington, Seattle, WA USA; 4 Vaccine and Infectious Disease Division, Fred Hutchinson Cancer Center, Seattle, WA USA; 5 Dermatology Branch, National Institute of Arthritis and Musculoskeletal and Skin Diseases, National Institutes of Health, Bethesda, MD USA; 6 Clinical Research Division, Fred Hutchinson Cancer Center, Seattle, WA USA; 7 Aprea Therapeutics, Philadelphia, PA USA; 8 University of Pennsylvania, Philadelphia, PA USA; 9 Present Address: University of Michigan, Ann Arbor, MI USA; 10 Present Address: Pacific Northwest University of Health Sciences, Yakima, WA USA

**Keywords:** Cancer, Immunology, Oncology

## Abstract

There is a major unmet need to develop therapies for patients whose tumors do not respond to PD-1 blockade. Inhibition of the ATR-mediated DNA replication checkpoint has been reported to augment antitumor immunity, although the underlying mechanisms remain poorly understood, particularly in cGAS-STING-deficient tumors. Here, we investigated the effects of ATR inhibition (ATRi) on tumor immunogenicity in Merkel cell carcinoma (MCC), an aggressive skin cancer commonly driven by Merkel cell polyomavirus (MCPyV). Among 92 primary MCC tumors, Ki-67 positivity was observed in 50% of MCPyV-positive and 83% of MCPyV-negative tumor cells, suggesting reliance on ATR-mediated replication stress responses. Notably, MCC frequently exhibits cGAS-STING deficiency, especially in MCPyV-positive tumors. In representative MCPyV-positive and -negative MCC cell lines, ATRi enhanced cytotoxicity and, alone or combined with low-dose radiation, induced proinflammatory NF-κB transcriptional programs. These included increased expression of MHC class I, antigen-processing machinery, interleukins, chemokines, and interferon-related genes. ATRi also increased surface calreticulin exposure and subsequent phagocytosis by human monocyte-derived macrophages. These findings support ATRi-mediated immune reprogramming in MCC cells through an NF-κB signaling cascade, regardless of cGAS–STING functional status. These observations support clinical evaluation of ATR inhibitors in immunotherapy-refractory disease (NCT05947500) and suggest candidate biomarkers of therapeutic response.

**Figure:**
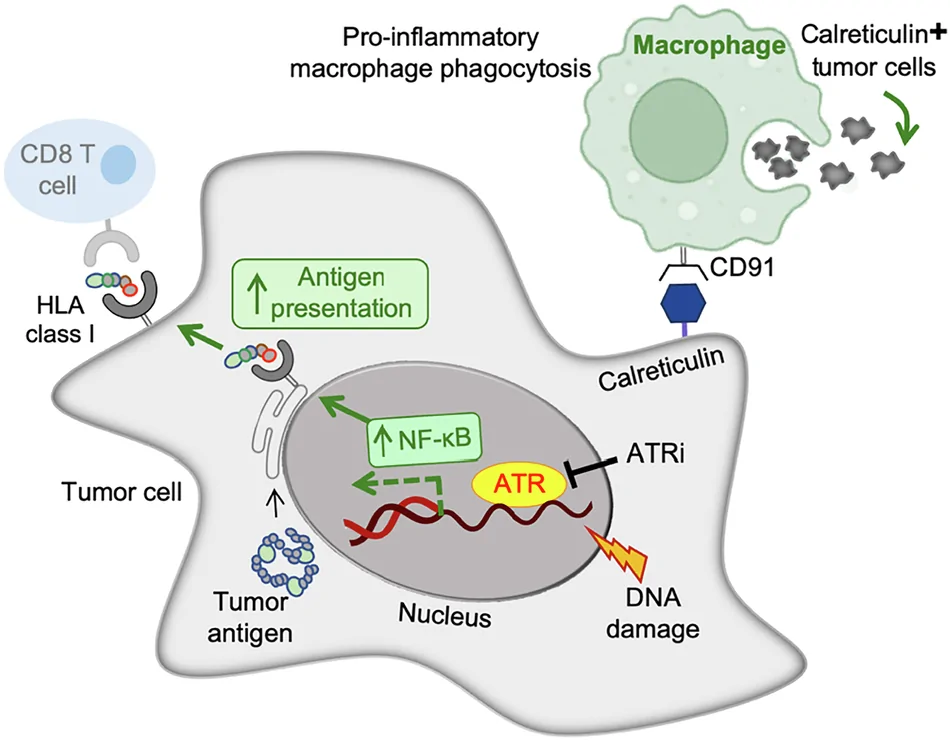

## Introduction

Immune checkpoint inhibitors (ICIs) have led to improved clinical outcomes for several advanced malignancies^1^. Malignancies that have proven particularly responsive to ICI therapies include melanoma^2^, Merkel cell carcinoma (MCC) and microsatellite instability-high tumors^3^. Response to PD-1 monotherapy can be as high as 60%, but durable response is below 50% in all cancers, in part due to acquired resistance. Underlying mechanisms of resistance can include tumor-cell-intrinsic (such as loss of class I major histocompatibility complex or MHC-I expression, defects in antigen processing machinery, etc.) and -extrinsic (e.g., absence of antitumoral M1-like macrophages, changes in tumor microenvironment via increased expression of T-cell exhaustion markers including PD-1)^4–6^. A standard second-line treatment for managing immunotherapy resistance in advanced, refractory melanoma and MCC is combining different ICIs to enhance immune response^1,5^. However, onset of Grade 3 or higher immune-related adverse events along with variable response (often low) across tumor types is a common challenge with ICI combination therapy^7–15^. Orthogonal approaches potentially with a wider therapeutic index could be useful in avoiding severe immune toxicity and promoting beneficial anti-tumor effects^1^.

Emerging evidence points to promising interactions between cell-cycle regulation and proinflammatory signaling, although the underlying mechanisms remain incompletely understood. Notably, the loss of early cell-cycle checkpoints renders rapidly proliferating tumors increasingly dependent on later cell cycle checkpoints, particularly the S and G2/M checkpoints, to preserve survival and genomic fidelity. A key coordinating protein in these later cell cycle checkpoints is the ATR (ataxia telangiectasia Rad-3 related) kinase^1^. ATR ensures completion of DNA replication prior to cell division and maintains genomic integrity through activation of replication stress checkpoints^16^. With the recent development of highly potent and selective ATR inhibitors (ATRi), several preclinical^17–22^ and clinical studies^23–28^ have revealed a surprising capacity for an ATRi to stimulate innate and adaptive anti-tumor immunity, particularly in combination with complementary modalities such as radiation or immune checkpoint blockade, largely through activation of the STING (stimulator of interferon genes)-interferon signaling axis^29^.

Given the significant unmet clinical need in the ICI-refractory setting^30^ and its inherent immunogenicity^1,31^, MCC represents a compelling testbed to evaluate the immunostimulatory potential of ATRi. MCC is a highly mitotic, rapidly growing malignancy characterized by a high fraction of tumor cells actively engaged in the cell cycle^32,33^. MCC tumors exhibit features consistent with substantial replication stress driven by disruption of key cell-cycle checkpoints, including retinoblastoma pathway inactivation, p53 pathway dysfunction, and elevated L-MYC expression^1,34^. These molecular aberrations are driven by Merkel cell polyomavirus (MCPyV) oncoproteins in ~80% of cases and by ultraviolet (UV)-induced mutations in the remaining 20% of patients in the United States^1,34^. Interestingly, MCPyV-positive MCC^35^, as well as other tumor types in advanced stages^36^, are associated with functional attenuation of the immunogenic cGAS (cyclic GMP-AMP synthase)-STING pathway. Here, we characterize tumor-intrinsic immune mechanisms augmented by ATR inhibition—administered alone or in combination with radiation—in both MCPyV-positive and -negative MCC cell lines. Collectively, our findings demonstrate that ATR inhibition augments nuclear factor kappa-light-chain-enhancer of activated B cells (NF-κB)-associated gene expression programs, potentially reversing immune resistance mechanisms relevant in MCC tumors^37,38^.

## Results

### ATRi induces cytotoxicity in MCC tumor cells

**Fig. 1:**
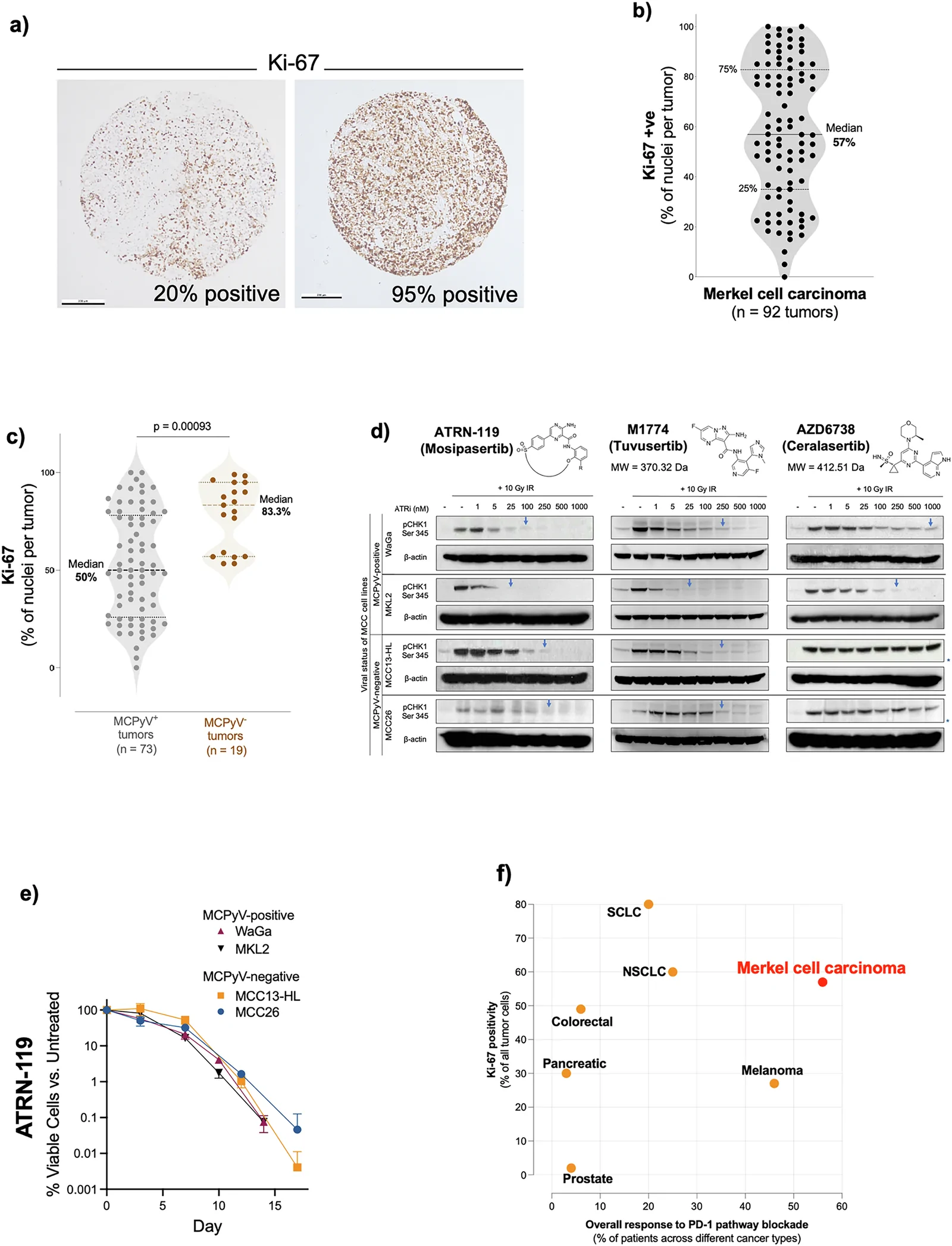
MCC tumor cells are highly proliferative and sensitive to ATR inhibition in vitro. **a** Illustrative IHC images showing 20% or 95% of nuclei positive for Ki-67 (indicates mitotically active cells) within Merkel cell carcinoma (MCC) patient tumor cores. **b** A scatter plot depicting the percentage of nuclear Ki-67 positivity from 92 unique MCC patients (four of the initially sampled 96 tumors had insufficient tissue for evaluation; Methods). Across the cohort, the median percentage of MCC tumor cells with Ki-67 positive nuclei was 57% (black dots across the scatter plot). **c** Ki-67 expression for Merkel cell polyomavirus (MCPyV)-positive (gray dots) and -negative (brown dots) tumors was 50% and 83%, respectively. The *p*-value, calculated using the Mann-Whitney test, is indicated. **d** Immunoblot analysis of p-CHK1 Ser 345 (the phosphorylation of cell-cycle checkpoint kinase 1 at Ser 345 residue) expression in representative MCPyV-positive (WaGa and MKL2) and -negative (MCC13-HL and MCC26) MCC cell lines treated with clinical-stage ATR (ataxia telangiectasia Rad-3 related) inhibitors ATRN-119 (mosipasertib), M1774 (tuvusertib), and AZD6738 (ceralasertib); ATRi. Drugs were administered one hour prior to 10 Gray of ionizing radiation. Whole cell lysates were collected after an additional three-hour incubation and analyzed for pCHK1 Ser 345 and beta-actin. *N* = 2. Blue arrows indicate the dose at which CHK1 Ser 345 phosphorylation was inhibited by >90%. *Effects on pCHK1 activity at AZD6738 concentration above 1 μM in MCPyV-negative MCC cell lines are detailed in Supplementary Fig. 1, but were at 4 μM for MCC13-HL and ~8 μM for MCC26. **e** Serial passaging with continuous ATRN-119 treatment (100 nM for MCPyV-positive and 250 nM for MCPyV-negative MCC; *N* = 3 per cell line) blocked viability of all cell lines relative to untreated cells of that line; as assessed by live-cell staining with trypan blue (WaGa maroon; MKL2 black; MCC13-HL yellow; MCC26 blue). **f** Across diverse tumor types, median Ki-67 positivity was plotted against responsiveness to an immune checkpoint inhibitor for the indicated cancer (effect size weighted by the number of patients in each cohort; see text for details).

We assessed the baseline proliferative index of MCC from 92 patient tumors using Ki-67 immunohistochemistry (IHC), a marker of cell proliferation. The median percentage of Ki-67-positive tumor cells per subject across this cohort was 57% (Fig. 1a, b). Strikingly, Ki-67 labeling varied markedly among MCPyV-positive and MCPyV-negative MCCs. Specifically, MCPyV-negative tumors exhibited a higher proliferative index (median Ki-67 positivity, 83%) compared to MCPyV-positive tumors (50%), Fig. 1c, consistent with the reported more aggressive clinical behavior of this subset^39^.

To determine if there are appreciable differences in ATR sensitivity across virus-positive and -negative MCC, we used three selective, orally bioavailable ATR inhibitors (ATRi). For each cell line, the potency of each compound to inhibit CHK1 (checkpoint kinase 1) phosphorylation at Ser 345, 3 h following ionizing radiation exposure (10 Gray) was assessed via western blot (Fig. 1d).

As expected, ATRN-119 and M1774 agents inhibited phosphorylated CHK1 or pCHK1 Ser 345 at lower concentrations, whereas AZD6738 treatment required higher doses to achieve a comparable loss of pCHK1 signal across the respective tumor cell lines. Of interest, MCPyV-positive cell lines were more sensitive to the effects of ATR inhibition than MCPyV-negative cells, requiring ~1.5–2-fold lower drug concentrations to achieve comparable pCHK1 inhibition. As ATRN-119 became available early in our studies and exhibited strong potency, it became the principal compound assessed in subsequent assays.

Even in the absence of exogenous DNA damage, continuous exposure of MCC cell lines (WaGa, MKL2, MCC13-HL and MCC26) to ATRN-119 over multiple cell passages resulted in markedly fewer viable tumor cells over time, Fig. 1e. Drug-induced cytotoxic effects on MCC cells were observed after approximately two weeks of treatment, suggesting the potential of ATRN-119 to demonstrate single-agent activity in both MCPyV-positive and -negative tumors.

Consistent with its high proliferative index and sensitivity to anti-PD-(L)1 therapy, MCC occupies a unique position relative to other cancer types when these variables are jointly considered^40–47^ (Fig. 1f), reflecting the uncommon coexistence of high tumor cell proliferation and intrinsic immunogenicity.

### ATRi enhances inflammatory NF-kB signaling, MHC-I and antigen presentation machinery genes

**Fig. 2:**
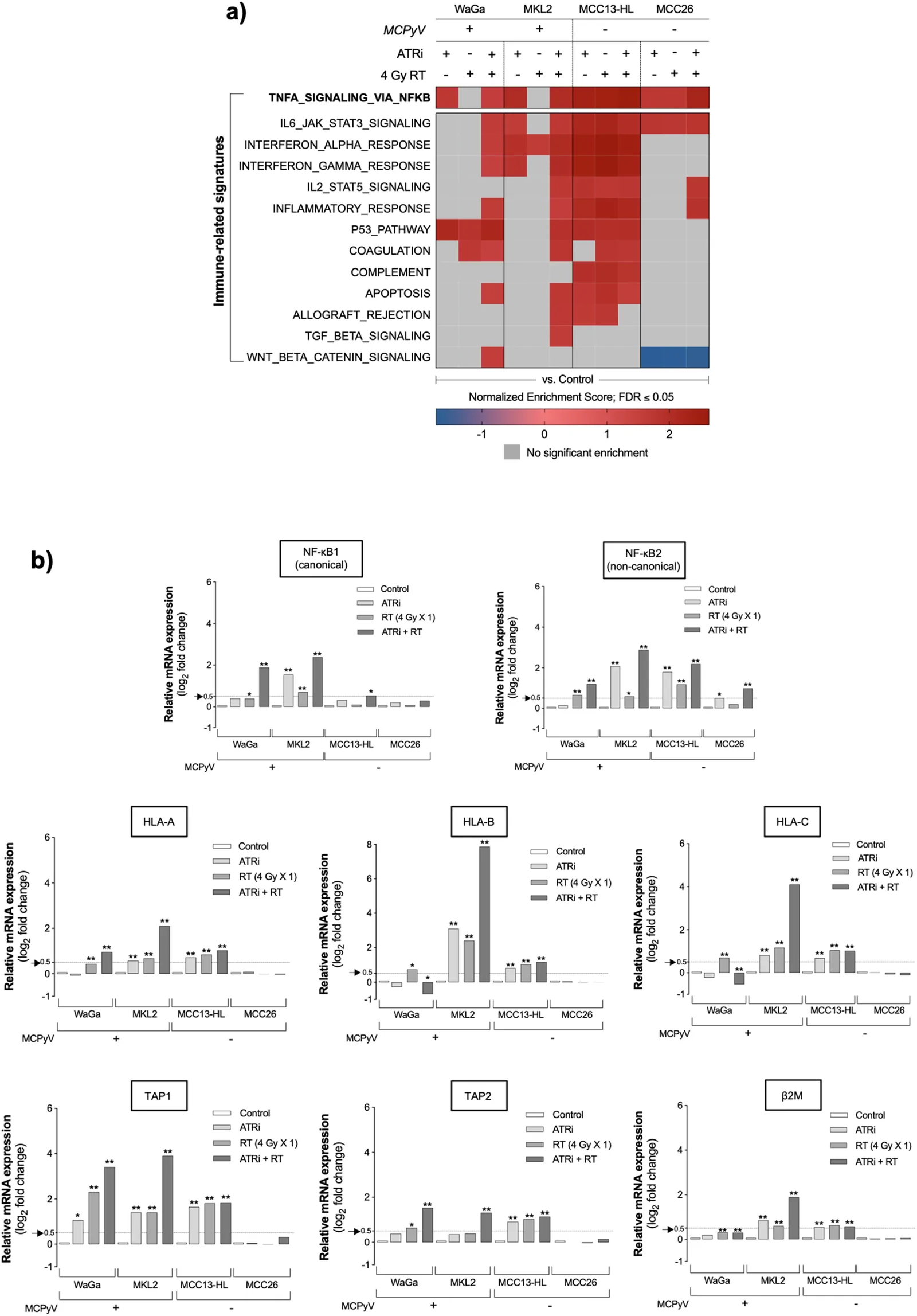
ATR inhibition enhances immune gene expression in MCC, including inflammatory NF-κB signaling, MHC-I and antigen presentation machinery-associated transcripts. **a** Four MCC cell lines were treated with ATRN-119 (100 nM for MCPyV-positive and 250 nM for MCPyV-negative MCC) one hour before radiation therapy (RT), as indicated (*N* = 3 per cell line). Cells were harvested for whole-transcriptome analysis of gene-drug response associations after 72 h. Data from treated versus control cells were analyzed through the human MSigDB GSEA (Molecular Signatures Database Gene Set Enrichment Analysis) pathway enrichment tool. Inflammatory pathways with significantly altered expression are represented using the colors indicated in the key (upregulated to downregulated: red to blue). The TNFα-NF-κB expression signature was induced in all MCC cell lines and corresponding gene set enrichment plots are included in Supplementary Fig. 3. **b** Effects of ATRi ± 4 Gy radiation on key pathways linked to tumor immune responsiveness. All data reflect bulk gene expression at 72 h after the indicated treatment. Gene expression changes following ATRN-119 ± 4 Gy RT treatment were considered statistically significant at log_2_fold change > 0.5 and Q ≤ 0.05 (* < 0.05; **<0.001), based on normalized transcript values. Graphical coding: ATRi + RT, dark gray; RT alone, light gray; ATRi alone, opaque; Control, transparent.

To investigate the ability of ATRi to enhance MCC immunogenicity, we used bulk RNA sequencing and assessed if proinflammatory gene signatures were enriched 72 h after treatment. The TNF-alpha hallmark gene set^48^ was strongly induced in all four cell lines, regardless of tumor viral status and did not require radiation for strong induction, Fig. 2a (Supplementary Figs. 2 and 3).

In addition, several other inflammatory pathways were variably activated by ATRi, with or without radiation, including interleukin-6, p53 and other cytokines. To better understand which transcripts drive these signatures, we analyzed the genes that were strongly induced. Figure 2b shows expression of highly influential individual transcripts that underlie the pathways depicted in panel 2a.

In particular, we were interested in the canonical (*NF-κB1*), non-canonical (*NF-κB2*) transcripts of NF-κB signaling as well as those involved in antigen processing and presentation, including human leukocyte antigens (*HLA-A, HLA-B, HLA-C*), the ATP-binding cassette transporters associated with antigen machinery (*TAP1* and *TAP2*), and β2-microglobulin (*β2M*). Across distinct treatment conditions and cell lines, we observed robust and statistically significant induction of the non-canonical NF-κB pathway-associated transcripts (NF-κB2 and RelB); Fig. 2b and Supplementary Fig. 4. With or without 4 Gy radiation, ATR inhibition promoted increased expression of multiple antigen-processing genes (Fig. 2b and Supplementary Fig. 4). One MCPyV-negative line (MCC26) was minimally affected by ATR inhibition with or without radiation in both inflammatory (Fig. 2a) and antigen-processing pathways (Fig. 2b).

### ATR inhibition induces immunostimulatory damage-associated molecular patterns

**Fig. 3:**
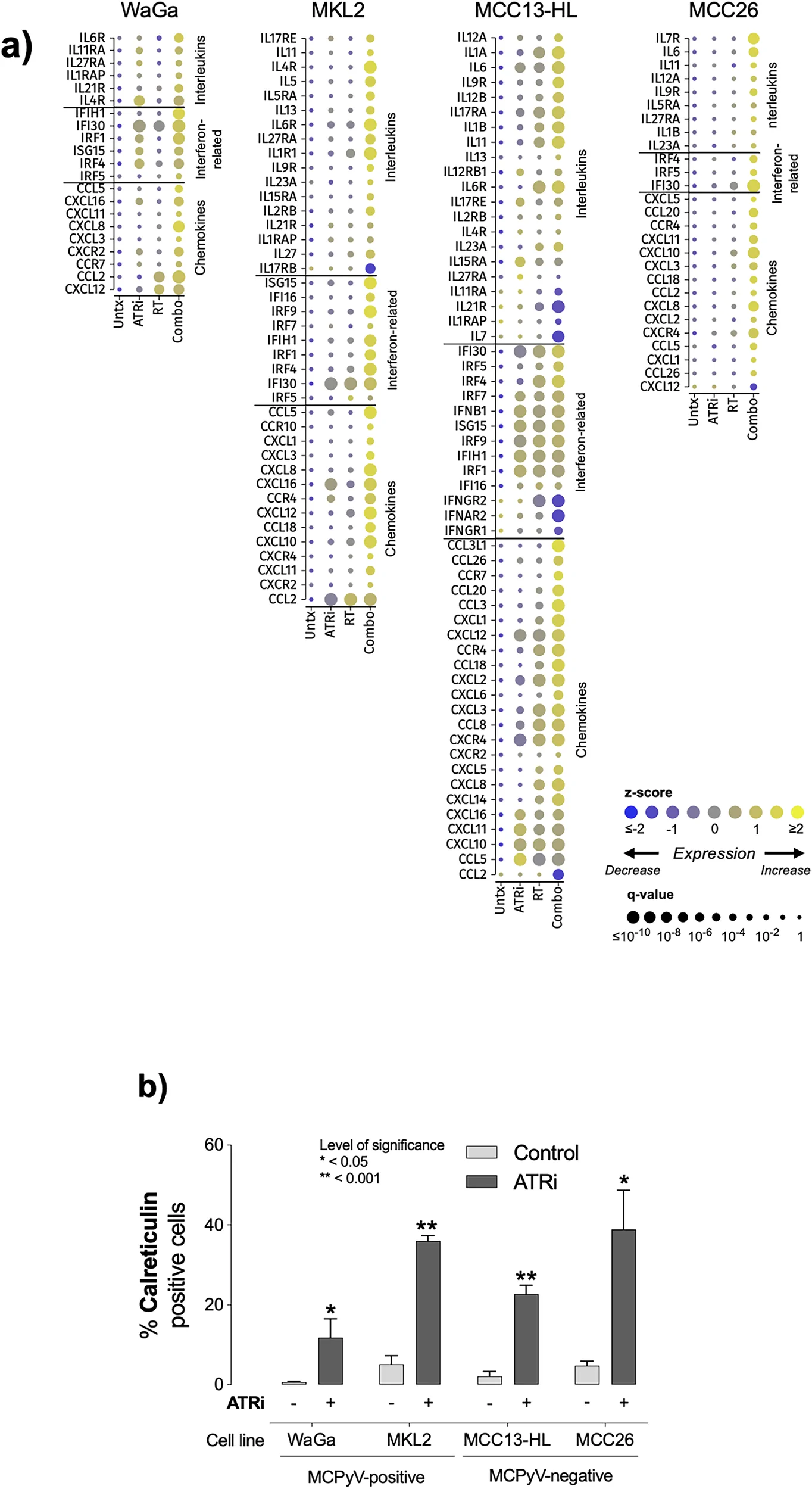
Inhibition of ATR stimulates immunostimulatory damage-associated molecular patterns^108^ in MCC. **a** Dot plot illustrating differential expression of immune-related transcripts grouped into three functional modules: interleukins, interferon-related genes, and chemokines, in MCC cell lines treated with ATRN-119 (100 nM for WaGa and MKL2; 250 nM for MCC13-HL and MCC26), with or without 4 Gy radiation, assessed at 72 h. Gene expression values from bulk RNA-seq dataset were normalized and z-transformed to allow comparison of relative transcript abundance across treatment conditions. Dot color represents the z-score, and dot size indicates statistical significance. The color scale ranges from purple (≤ -2, lower expression) to yellow (≥ +2, higher expression), highlighting variation in gene expression across samples. The majority of genes represented are pro-inflammatory mediators commonly associated with immune activation downstream of immunogenic cell death. **b** Flow cytometry analysis of surface calreticulin expression at Day 12 in control versus ATRN-119-treated MCC cells, dosed as above. The percentage of calreticulin-positive cells, quantified from flow cytometry (*N* = 3), is shown in representative histograms; Supplementary Fig. 6. Statistical significance was estimated through multiple unpaired t-tests. Treatment groups: ATRi, dark gray, Control, light gray.

In the dot plot depicted in Fig. 3a, early, proximal signaling responses to ATR inhibition in individual proinflammatory chemokine, interferon-related and interleukin genes were profiled via bulk transcriptome sequencing across each cell line and treatment condition at 72 h. Individual statistically significant transcripts are shown in descending order based on the magnitude of gene expression change promoted by the combination treatment in both MCPyV-positive and MCPyV-negative characteristic disease models.

For a majority of these genes, the combination of ATRi plus RT elicited substantially greater changes in gene expression compared to either treatment alone. As in Fig. 2, gene induction was weakest in the MCC26 virus-negative cell line. While there was substantial overlap in the cytokines induced across cell lines, the strength of induction for specific genes varied appreciably with each treatment exposure at the 72 h timepoint.

Figure 3b shows that calreticulin, a hallmark of immunogenic cell death, was markedly upregulated by Day 12 of ATRi monotherapy exposure as compared to untreated cells.

### ATR inhibition, alone or with low-dose radiation, enhances phagocytosis of MCC tumor cells by human macrophages

To determine whether increased expression of calreticulin on MCC tumor cells corresponded to functional engulfment by macrophages, we used a standard in vitro fluorescent dye-based engulfment assay (Supplementary Fig. 7a).

**Fig. 4:**
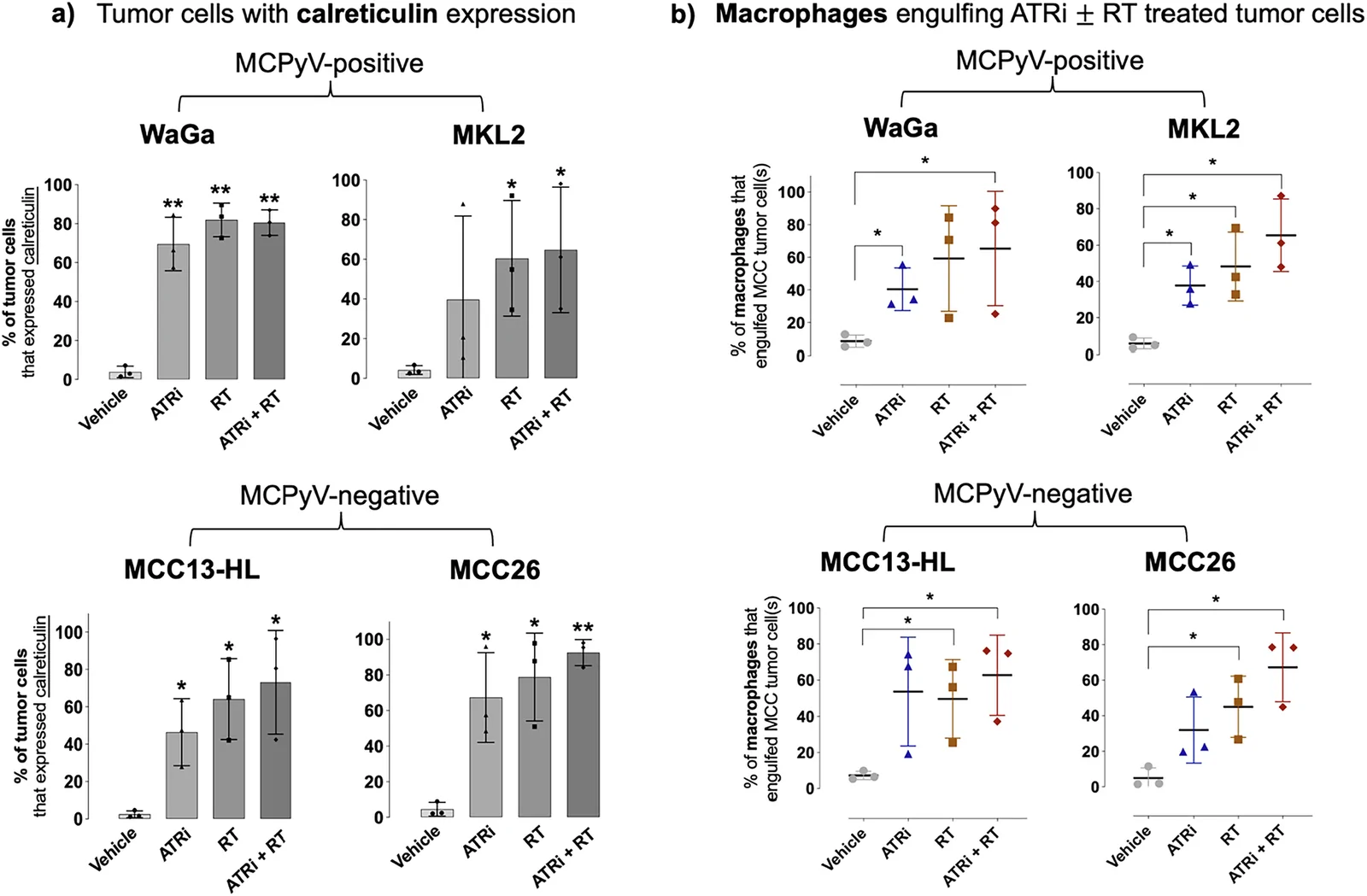
ATR inhibition, alone or with low-dose radiation, enhances phagocytosis of MCC tumor cells by human macrophages. Representative MCC tumor cells were treated with ATRN-119 (100 nM for WaGa and MKL2; 250 nM for MCC13-HL and MCC26) ± 4 Gy RT. 72 h later, tumor cells were labeled (pHrodo dye) and then co-cultured for ~1 hour with activated human macrophages (derived from peripheral blood mononuclear cells, Supplementary Fig 7). **a** Surface calreticulin expression on treated virus-positive and –negative MCC cells is shown as percent positive per treatment condition. Bar shading indicates treatment group: Vehicle (white), ATRi (light gray), RT (medium gray), and ATRi + RT (dark gray). **b** The percentage of macrophages that had engulfed at least one pHrodo-stained MCC cell(s) is indicated. Unpaired two-tailed t-tests were performed between each treatment and the control group, without correction for multiple comparisons (*<0.05; **<0.001); *p*-values for vehicle vs ATRi, RT, ATRi + RT; WaGa = 0.0155, 0.0544, 0.0493; MKL2 = 0.0079, 0.0189, 0.0069; MCC13-HL = 0.0559, 0.0283, 0.0126; MCC26 = 0.0743, 0.0187, 0.0059. Simultaneous phenotypic characterization of macrophages was performed, including expression of the calreticulin (CRT) receptor, human CD91, as well as CD68 and CD80 (Supplementary Fig. 7b and 7c); CD denotes cluster of differentiation. Treatment groups are distinguished by color and symbol as follows: Vehicle (gray circles), ATRi (blue triangles), RT (gold squares), and ATRi + RT (red diamonds).

Briefly, human monocyte-derived macrophages were co-cultured with treated (ATRi ± RT) tumor cells that had been labeled with the pH-sensitive pHrodo dye and flow cytometry was used to measure tumor cell engulfment. Treatment with an ATR inhibitor, radiation, or the combination robustly induced calreticulin surface exposure, a pro-engulfment ‘eat-me’ signal, after 72 h in both MCPyV-positive and -negative cell lines (Fig. 4a).

Indeed, co-culture with human monocyte-derived macrophages revealed significantly increased macrophage engulfment of pre-treated MCC tumor cells (Fig. 4b). Of note, across all cell lines tested, combined exposure to ATR inhibition and radiation was more potent in stimulating tumor cell engulfment than either treatment alone.

### Transcriptomic correlation between replication stress and inflammatory response

**Fig. 5:**
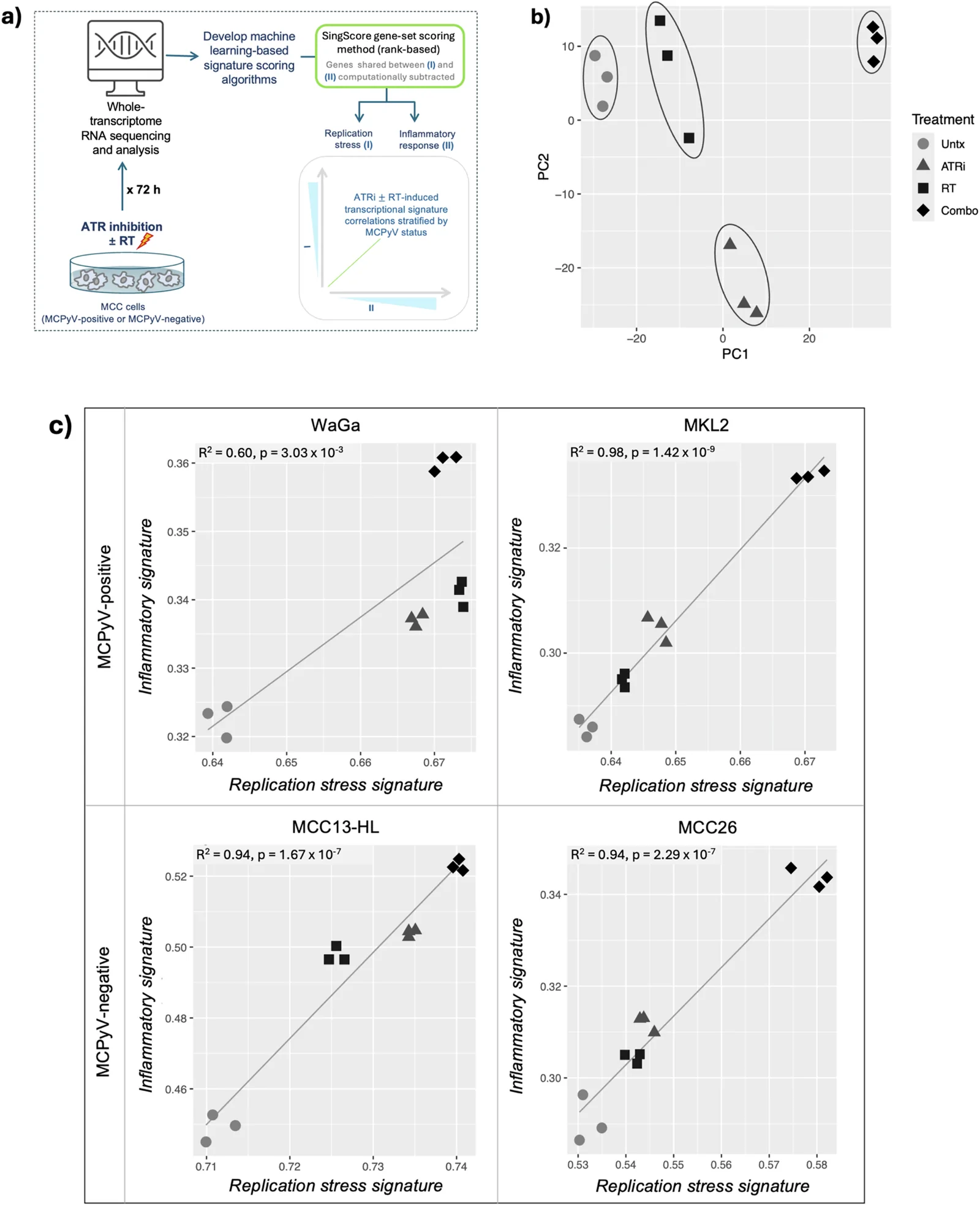
Transcriptional responses including replication stress and tumor-intrinsic inflammation following ATRi ± radiation. **a** Differentially expressed genes from bulk RNA-seq-derived datasets were evaluated per cell line for curated replication stress- and inflammatory response-associated transcriptional signatures. Selected MSigDB hallmark gene sets, as outlined in the Methods, were assigned to each category represented on the x- and y-axes. Treatment conditions: WaGa and MKL2 100 nM ATRN-119; MCC13-HL and MCC26 250 nM ATRN-119, with or without 4 Gy RT, for 72 h. **b** Normalized bulk RNA-seq data were analyzed through principal component analysis (PCA), a linear dimensionality reduction method, to depict the transcriptional space of each treatment group, with data combined across the four cell lines. The four treatment conditions induced transcriptional states that were distinct from each other. **c** Transcriptomic data were stratified by cell line and treatment as indicated (*N* = 3 biological replicates per treatment per cell line). Replication stress and tumor-intrinsic inflammation signatures were derived and analyzed using GSEA MSigDB hallmark pathways. Clustering patterns and coefficients of determination (R^2^) varied by treatment across cell lines as indicated. Notably, for each cell line, the combination of ATR inhibition and radiation more strongly induced these hallmark pathways relative to either treatment alone; Supplementary Fig. 9. Treatment groups are denoted by symbol: Untreated (circles), ATRi (triangles), RT (squares), and ATRi + RT (diamonds).

To assess aggregate treatment effects, bulk RNA-sequencing data were analyzed for global transcriptional responses across four cell lines (Fig. 5a). As shown in Fig. 5b, the indicated treatments led to changes in transcription linked to replication stress and inflammation across the four cell lines (WaGa, MKL2, MCC13-HL, and MCC26). The greatest effect was seen with combined ATR inhibition and radiation.

Figure 5c depicts correlations between replication stress and inflammatory transcriptomic signatures induced by ATR inhibition ± radiation. Transcriptomic data for each cell line were queried against selected MSigDB hallmark pathways to generate quantitative signature scores for replication stress and tumor-intrinsic inflammation (see Methods). Within each cell line, the combination treatment most effectively augmented responses along both axes; distinct clustering patterns for each treatment condition and strong goodness-of-fit across etiology were noted (R^2^: WaGa = 0.60, MKL2 = 0.98, MCC13-HL = 0.94, MCC26 = 0.94). The linear relationship observed between the two signatures across treatment conditions, relative to control, emphasizes the relevance of concurrent transcriptionally regulated replication stress and immune reprogramming features that were temporally augmented following ATR inhibition, with or without radiation.

## Discussion

To improve outcomes with immune checkpoint blockade in patients with primary or acquired resistance, several therapeutic classes are being investigated in preclinical and clinical settings in MCC and other solid tumors. Such approaches include ICI combinations^13,49^, immune-sensitizing targeted therapies^50^, gut commensals and their metabolites^51^, antibody-drug conjugates^52^, and epigenetic modifiers^53,54^. Notably, there is growing evidence linking cell cycle biology, CDK4/6 inhibition in particular, with anti-tumor immunity^55,56^. Therapeutic strategies targeting DNA damage response mechanisms, such as PARP (poly [ADP-ribose] polymerase)^57^, ATR^29^, ATM (ataxia telangiectasia mutated)^58^, Chk1^59^, DNA-PK (DNA-dependent protein kinase)^60^, and Wee1^61^, are being evaluated in various combinations for synergistic immune-enhancing effects.

A key mechanism connecting cell-cycle defects and immune sensitization is activation of the intracellular DNA-sensing cGAS-STING pathway^62–65^. cGAS-STING can promote innate immune recognition of tumors but is variably functional across tumor types^35,36,66^ and tends to be inactivated in more advanced / metastatic disease. Moreover, the cGAS-STING signaling pathway is defective in MCC tumors driven by the Merkel cell polyomavirus (MCPyV)^35,67^.

In this study, we explored tumor-intrinsic immune mechanisms promoted by potent and selective ATR inhibitor(s), with or without low-dose radiation, in MCPyV-positive and -negative cell lines. Strikingly, treatment with an ATRi reversed transcriptional downregulation of HLA class I and antigen processing genes^37^, alongside induction of the TNF-NF-κB hallmark signature across all four tested MCC cell lines. Simultaneously, there was increased surface expression of the pro-phagocytic calreticulin signal on treated tumor cells that corresponded to increased engulfment by activated human macrophages.

ATR, a member of the serine / threonine phosphatidylinositol-3 kinase-related kinases family, has long been recognized as a promising therapeutic target in cancer, particularly for sensitizing G1 checkpoint-deficient cells to DNA replication stress^68–70^. The recent development of highly specific and potent small-molecule inhibitors has enabled new insights regarding the capacity of ATR inhibition to enhance anti-tumor immune responses^71,72^. Augmented anti-tumor immunity via an ATRi has been observed in both preclinical and clinical settings^18,21–23,27,28,71,73^. This anti-tumor immune effect potentially extends the clinical benefit of ATR inhibition beyond merely sensitizing tumor cells to chemotherapy and or DNA-damage^1^.

Given its high proliferation index and frequent responsiveness to ICI agents (Fig. 1f), MCC represents a relevant disease in which to study the intersection of cell-cycle regulation and immune checkpoint biology^1^. When combined with palliative radiotherapy or ICIs in advanced human solid tumor specimens studied, ATR inhibition has been shown to modulate adaptive immune components, associated with upregulation of the TNF-NF-kΒ hallmark signature genes^27,28^. Furthermore, in patients with ICI-refractory melanoma, responses to ATRi plus anti-PD-L1 were more likely in patients whose baseline tumor samples showed a more inflamed tumor microenvironment characterized by enhanced MHC-I / antigen-presentation machinery expression^23^.

Nonetheless, preclinical evidence remains limited, and, in some contexts, controversial, regarding how ATR^74,75^ or its downstream effector Chk1^76,77^ may be involved in inflammatory signaling, such as NF-kB activation. Moreover, the cGAS-STING signaling mechanism is compromised in virus-positive MCC^35,67^. This pathway is believed to be important in the ability of an ATRi to induce innate immune activation signals in various cancer types^18,19,29,78–80^. Of the four cell lines tested here, the two virus-positive MCC cell lines have previously been shown to be defective in cGAS-STING activity while the virus-negative lines are intact in this pathway^35,67^. Indeed, our own studies of these 4 cell lines corroborate the prior findings, and an overview of the data regarding tumor-intrinsic cGAS–STING function for these lines is provided in Supplementary Table 1. Therefore, in the present study, we were able to assess the ability of an ATRi to augment anti-tumor immunity in the presence and absence of a functional cGAS-STING pathway.

We found that treatment with an ATRi consistently induced the TNF-NF-kB signaling gene set in all four lines, a hallmark of proinflammatory activation. These findings are consistent with recent studies showing that ATR inhibition augments TNF-NF-kB signaling both in vitro for cell lines of other cancer types^75^ and in human clinical trials^27,28,73^. Interestingly, ATR inhibition—either as monotherapy or in combination with palliative irradiation—has been associated with upregulation of TNF-NF-kB-associated intratumoral inflammation in advanced solid tumors. Indeed, this signature was enriched at baseline among ATRi-responders in two independent clinical studies^28,73^. More specifically, transcriptional induction of non-canonical NF-kB2 (Fig. 2) and its substrate RelB (Supplementary Fig. 4), together with IFI30 (Interferon-gamma-inducible protein 30) and ISG15 (Interferon-Stimulated Gene 15), emerged as prominent ATRi ± RT-induced immune markers in MCC.

Other studies have recently shown that NF-kB axis activation promotes the expression of class I MHC and proteins required for antigen presentation (e.g., TAP1, TAP2 etc.)^74,81–85^. These convergent findings motivated us to explore the extent to which NF-kB signaling is required for the ability of an ATR inhibitor to promote expression of antigen presentation machinery. Strikingly, co-treatment with an NF-kB inhibitor attenuated—and in some cases reversed—the ATRi ± RT-driven increase in antigen-processing pathway proteins in MCC tumor cells (MCPyV+ and MCPyV- cell lines, with periodic authentication by short tandem repeat (STR) profiling; Supplementary Fig. 5a and 5b), congruent with transcriptional expression at 72 h (Fig. 2 and Supplementary Fig. 4). Although interactions between NF-kB and cGAS-STING are incompletely understood, NF-kB blockade reduced baseline cGAS abundance in the MCC13-HL tumor cells (MCPyV-negative; Supplementary Fig. 5b). Consistent with this, the NF-kB inhibitor also led to reduced STING activation followed by reduced IRF3 phosphorylation in these virus-negative, cGAS-STING-proficient cells^35^; Supplementary Fig. 5b. These early data help isolate the effects of NF-κB modulation on intracellular antigen processing and type I IFN signaling^86^ induced by ATR inhibition, either alone or in combination with low-dose radiation, independent of baseline cGAS-STING activity.

Alongside, simultaneous attempts to quantify class I MHC surface expression after 72 h of combination treatment with ATRi and low-dose radiation resulted in substantial loss of cell viability. However, these observations are difficult to interpret in the context of the concurrent activation of cytotoxic mechanisms and treatment-induced cell death, which may have confounded assessment of cell-surface HLA-ABC expression at this timepoint (data not shown).

In addition to its role in tumor-intrinsic innate immunity, its effect on the TME, and T cell activation, the NF-kB pathway is a critical determinant of M1 macrophage polarization in response to inflammatory stimuli—including damage-associated molecular patterns such as calreticulin^87–91^. These observations are consistent with findings that indicate NF-kB and pro-phagocytic calreticulin expression are associated^89^ with augmented tumor cell phagocytosis in vitro. Together, these data support a model in which NF-kB—independent of inducible cGAS-STING function—plays an important role in the proinflammatory, anti-tumor responses elicited by therapeutic ATR targeting.

While the current ex *vivo* studies enable controlled investigation of signaling mechanisms relevant to anti-tumor immunity, they do not permit direct assessment of complex tumor-immune interactions such as engagement with antigen presenting cells, T-cell recognition, or immune cell infiltration within the TME. Additionally, bulk RNA-seq captures averaged gene expression across heterogeneous cellular states and may not adequately predict patient-level response, particularly in heavily pre-treated cohorts.

In this context, and consistent with U.S. Food and Drug Administration (FDA)-recognized New Approach Methodologies^92^, this work leverages human-relevant, fit-for-purpose experimental systems to address key translational gaps between conventional preclinical models and clinical outcomes. Specifically, these studies aim to define early, tumor-intrinsic mechanistic features of ATR inhibitor-mediated immune re-sensitization, either as monotherapy or in combination, in a rare and highly proliferative malignancy of the skin for which representative in vivo models remain limited.

In metastatic MCC with primary or acquired resistance to immunotherapy, the multi-center, randomized Phase II MATRiX study evaluated ATR inhibition alone and in combination with anti-PD-L1 therapy (NCT05947500). ATRi monotherapy did not yield objective responses among the first 10 treated patients, despite preclinical evidence of efficacy in settings with intact cGAS-STING signaling^29,93^. In contrast, the addition of avelumab led to clinically meaningful tumor regressions in a subset of ICI-refractory MCC patients, supporting continued enrollment to 25 patients in the combination therapy arm^94^. Consistent with these findings, ATR inhibitor monotherapy has demonstrated relatively little efficacy across multiple tumor types, including those harboring DNA damage response (DDR) alterations predicted to confer ATR dependency^95,96^.

Intriguingly, the markedly elevated proliferative index observed in virus-negative MCC suggests an increased burden of replication stress in this subgroup. It will be interesting to assess in ATRi plus avelumab-responsive cases whether those tumors had particularly elevated Ki-67 fractions.

Multiple ATRi compounds are being assessed as radiotherapy combinations across diverse clinical settings, with some reports of striking anticancer activity^28,71^ (NCT05566574, NCT04576091, NCT04052555, NCT02589522). Given the well-established radiosensitivity and immunogenicity of MCC^1,97^, ATRi may synergize with radiation and immune checkpoint blockade to heighten tumor immunogenicity and reduce disease burden in advanced, ICI-refractory patients with MCC. Of note, the triple combination (ATRi + radiation + ICI) approach has reliably been associated with enhanced adaptive immunologic memory and improved survival in murine models^19,22,25,98^.

Accumulating data also underscore the importance of timing ATRi treatment relative to radiation. Indeed, it is important for the ATRi to be present at the time of, and likely for around 24 h after RT. However, the continued presence of ATRi for up to or beyond a week following RT is associated with diminished expansion and function of tumor-specific T cells that presumably proliferate rapidly in response to the immunogenic tumor cells following RT + ATRi^21,98^. Indeed, compared with continuous ATRi therapy, intermittent ATRi dosing is more effective in synergizing with radiation to promote anti-tumor immunity in mouse models^21,98^.

Nevertheless, the clinical development of DDR-targeting agents remains challenging^71,99^, owing to difficulties in measuring consistent target engagement^100^, uncertainties around optimal dosage and frequency, the need to identify tumor type(s) most likely to benefit, and the complexities inherent to the multitude of rational combinations such as radio-immunotherapy and pairings with cytotoxic or DDR-based therapies. As discussed, ATRi monotherapy has shown limited efficacy in DDR-deficient tumors, reinforcing the need for dual or triple-agent combinations to enhance tumor priming and therapeutic susceptibility.

There are encouraging signals for ATRi in some settings, such as the recent FDA Fast Track designation of alnodesertib (formerly ART0380^101^, in combination with low-dose irinotecan) as a third-line treatment for ATM-negative metastatic colorectal cancer (NCT04657068^102^). This regulatory designation showcases the promise of next-generation ATR-targeting strategies and reinforces their therapeutic potential in difficult-to-treat settings.

In summary, beyond its sensitizing effects in tumor cells deficient in early cell-cycle-checkpoint^68^, ATRi therapy has the potential to synergize with radiation and immune checkpoint blockade. This work provides additional mechanistic insight into the optimal design of future trials that attempt to leverage ATRi-mediated enhancement of tumor immunogenicity.

## Methods

### Ki-67 immunostaining in MCC tumors

Tissue specimens from 96 patients with MCC were collected following written informed consent and approval by the Fred Hutchinson Cancer Center Institutional Review Board (IRB #6585). All related biological procedures were conducted in compliance with the Declaration of Helsinki guidelines and its subsequent amendments. For the 92 subjects with evaluable tumor specimens, triplicate 0.6-mm cylindrical cores from formalin-fixed, paraffin-embedded donor tumor blocks were arrayed onto charged slides for tissue microarray (TMA) construction. After heat-induced epitope retrieval with Dako Citrate Buffer (pH 6.0; 40 min), slides were blocked with TCT buffer (0.05 M Tris, 0.15 M NaCl, 0.25% casein, 0.1% Tween-20) solution for 30 min. Nuclear Ki-67 staining was performed using the MIB-1 primary antibody (M7240, Dako; 1:100) on an automated stainer for 60 min, followed by incubation with secondary antibody (Biocare Mach2 anti-mouse HRP) for 30 min at ambient temperature. Tonsil cores served as positive controls. Signal detection was achieved using 3,3′-diaminobenzidine chromogen, with hematoxylin and eosin counterstaining for morphological assessment.

Brightfield imaging and evaluation were performed by trained pathologists, followed by scoring of immunostained slides for signal intensity based on the percentage of tumor nuclei positive for Ki-67. Ki-67 scores were averaged across multiple tissue cores per case, and the median percentage of positive nuclei per tumor was plotted. Quantitative assessment of the Ki-67 labeling index was additionally performed, stratified by MCPyV status^39^, with median values reported for each group.

### Cell lines and culture conditions

MCPyV-positive WaGa and MKL2 suspension cells were received as a kind gift from Jürgen Becker (University of Duisburg-Essen, Germany) and Masahiro Shuda (University of Pittsburgh, USA) labs, respectively. Adherent MCC26 and MCC13-HL virus-negative cells were obtained from Helen Leonard (University of Western Australia, Australia) and Denise Galloway (Fred Hutchinson Cancer Center, USA), respectively. Cell lines were authenticated by STR profiling upon receipt (via American Type Culture Collection, USA), cryopreserved shortly thereafter, and periodically reauthenticated against reference profiles. Experiments were performed using periodically revived aliquots maintained for no more than 20–25 passages. All MCC cell lines were cultured in complete R-10 medium (10% fetal bovine serum, 2 mM glutamine and 1% penicillin-streptomycin). Cells were maintained at 37 ^o^C in a humidified incubator supplied with 5% CO_2._ Each cell line was routinely monitored for mycoplasma contamination (InvivoGen MycoStrip, rep-pt1).

### Compounds

AZD6738 (AstraZeneca, Cambridge, UK; Selleck Chemicals, S7693), was reconstituted in DMSO per manufacturer instructions. ATRN-119 was obtained from Aprea Therapeutics (previously ATRIN Pharmaceuticals) and was reconstituted in water to a 5 mM stock. M1774 (EMD Serono, Darmstadt, Germany) was solubilized in DMSO (10 mM stock).

### Immunoblotting

On Day 0, representative MCPyV-positive (WaGa and MKL2; ~3 ×10^6^ cells per well) and -negative (MCC26 and MCC13-HL; ~1.0 or 1.2 × 10^6^ cells per well) MCC cells were seeded in 6-well tissue culture plates. After 24 h, concentrations ranging from 1 nM to 1 μM were tested for each ATRi per cell line. Cells were exposed to the indicated ATRi for 1 h before 10 Gy of ionizing radiation treatment using X-ray biological irradiator (Rad Source Technologies RS2000, Georgia, USA) to activate immediate cellular repair mechanisms. Treated cells and vehicle control cells were collected, with mild-trypsinization for adherent cell lines or without trypsinization for non-adherent cell lines, 3 h post-irradiation under standard conditions. Harvested cells were washed with ice-cold 1X PBS and lysed in protein extraction buffer (150 mM NaCl, 1.0% IGEPAL CA-630, 0.5% sodium deoxycholate, 0.1% SDS, 50 mM Tris at pH 8.0 plus 1X protease-phosphatase inhibitor; Cell Signaling Technologies #5872). Next, soluble whole cell fraction lysates were isolated via centrifugation at 13,000 rpm for 10 min at 4^o^C and stored at −80^o^C until further use. Protein concentration was determined using Bradford assay. Normalized samples were resolved under reducing conditions using NuPAGE Bis-Tris gels and transferred to PVDF (polyvinylidene fluoride) membranes for immunoblot analyses. Membranes were probed with phospho-CHK1 Ser345 primary antibody (clone 133D3 1:1000 dilution; Cell Signaling Technologies #2348) overnight at 4^o^C, followed by expression detection with peroxidase-coupled secondary antibody (1:2500; Cell Signaling Technologies #7074). Blots were visualized by enhanced chemiluminescence (Bio-Rad Clarity Max Western ECL Substrate #1705062) captured on X-ray films or a digital imager. β-actin was used as internal loading control. Treatment conditions for Supplementary Fig. 5B, which summarizes the exploratory evaluation of NF-κB-induced antigen-processing machinery, including TAP1 and TAP2, tapasin, β2M, following ATR inhibition alone or in combination with radiation, are described in the corresponding figure legend. Each individual lane shown in Supplementary Fig. 5B was generated using the same protein extraction and immunoblotting protocol described above, with antibody dilutions optimized according to the manufacturer’s instructions, as applicable. All target antibodies were procured from Cell Signaling Technologies.

### Serial passaging and drug sensitivity assessment

MCPyV-positive (WaGa and MKL2; 300,000 cells per ml) and -negative MCC cell lines (MCC13-HL and MCC26; 100,000 cells per ml) were seeded in 6-well plates in R-10 growth medium. Cells were treated continuously with ATRN-119 (100 nM for WaGa and MKL2; 250 nM for MCC13-HL and MCC26). Cultures were passaged every 3-4 days. Control cells were maintained at ~50% confluency by 3–10-fold dilution twice weekly, and treated cells were diluted by the same factor at each passage. Drug-containing media was replenished at each passage at the indicated concentrations.

At defined intervals, viable cells were quantified at each passage using trypan blue exclusion. Percentage change in viable cells was calculated by normalizing the mean cell count of treated cells to untreated controls at each passage (in triplicate). Data were visualized using GraphPad Prism.

### Flow cytometry

At Day 12 ( ± 1 day) of ATRN-119 treatment, serially passaged virus-positive and -negative MCC cells were analyzed for cell-surface immune marker induction (*n* = 3 per condition). Adherent cells were washed once with ice-cold PBS and then enzymatically dissociated using 0.25% trypsin-EDTA (37^o^C for < 3 min) with quenching. Suspension cells were homogenized using gentle pipetting and collected in 5 ml FACS tubes. Mock-treated cells were used as baseline control.

Prior to staining for surface antigen expression across individual assays, harvested cells were washed in 1X PBS at 300xg for 5 min. Cells were incubated with Fixable Viability 780 dye (BD Biosciences #565388) for 15 min at room temperature, and the subsequent washing steps were carried out in staining buffer (BD Biosciences # 554657). Next, cells were stained at room temperature for 30–45 min with a cocktail of anti-human fluorochrome-conjugated monoclonal antibodies (mouse calreticulin Alexa Fluor 647, R&D systems #IC38981R; mouse HLA-ABC PE-Cy7; BD Pharmingen #561349). After 3 washes with wash buffer (BD Biosciences # 554723), at least 10,000 events per sample were acquired on BD FACSCanto (CantoRuo #641528). Unstained cells were used as technical controls to set up signal thresholds. Gating strategy is included in the Supplementary section. The fluorescence spillover was compensated for with individual single stain antibody-capture ( ± anti-mouse kappa beads, BD Biosciences #552843) or amine-reactive beads ( ± Arc beads, Invitrogen #A10346). Data were analyzed using FlowJo Version 10.9.0 and GraphPad Version 10.0.3 software. As part of FACS analyses, debris and doublets were excluded using forward scatter vs. side scatter parameters

### Bulk RNA sequencing and analysis

Early passaged WaGa, MKL2, MCC26 and MCC13-HL cell lines were used (sub-cultured less than 25 times). 72 h of ATRN-119 (100 nM for MCPyV-positive and 250 nM for MCPyV-negative) ± 4 Gy radiation (sub-lethal DNA damage to support mechanistic investigations in MCC) treatment was initiated in triplicate for cells seeded in 10 cm tissue-culture dishes. Untreated cells were used as negative control.

Harvested cells were submitted for RNA extraction, library preparation and sequencing (DNBseq platform, BGI Genomics, Cambridge, MA, USA). Raw paired-end reads ( ~ 30 million per sample; 100 base pair read length) were quality-checked, adapter-trimmed, and aligned to GRCh38 with Bowtie2^103^. Genes with counts less than 10 across all samples were filtered out. The subsequent raw RNA-Seq count data were normalized using FPKM (fragments per exonic kilobase per million), with effective library sizes estimated using TMM (trimmed mean of M-values), followed by log_2_ transformation with a pseudocount of 2, as implemented in the edgeR R/Bioconductor package^104^ and used for further analyses. Differential analysis at the gene level was performed using the Limma package^48^, with false discovery rate method used to adjust the *p*-values for multiple testing. Genes with an absolute log_2_ fold change of at least 0.5, normalized against mock-treated cells, were considered either up or downregulated and used for relative quantification across different treatment groups. Statistical significance was defined by a Q value of ≤ 0.05. Dot plots were generated using the R ggplot2 package^105^. Transformed expression data matrices were uploaded to an integrative cloud-based multi-omics RNA-seq data mining browser from BGI Genomics (“Dr. Tom”) and then used as input for computation of treatment-induced transcriptomic enrichment via the Molecular Signatures Database (MSigDB) hallmark gene set analysis. Hallmark interpretations were queried using a minimum of 15 and a maximum of 500 genes per set for statistically significant signatures. Distribution of normalized enrichment scores (NES), gene set enrichment analysis (GSEA)-defined significance at a false discovery rate (FDR) ≤ 0.05, and leading-edge (LE) gene set sizes for a characteristic biological phenotype is shown in Supplementary Fig. 3. Variable clustering of gene expression data across all samples was performed and visualized through principal component analysis.

### Correlation between replication stress and inflammatory signatures

A curated Replication Stress Signature was created using GSEA MSigDB programs comprising genes involved in DNA damage response and cell-cycle regulation pathways (including the Hallmark DNA repair, G2M checkpoint, and p53 Pathway gene sets), whereas the Inflammatory Signature was informed by immune network genes contained within the MSigDB Inflammatory Response category. Relevant differentially expressed genes for each treatment group, compared with untreated cells in the whole-transcriptome dataset, were used to construct treatment-specific gene signature lists for subsequent scoring. To generate the Replication Stress gene list, differentially expressed genes from the DNA repair, G2M checkpoint, and p53 hallmark gene sets were selected and retrieved from the MSigDB Hallmark database^106^. Similarly, among the differentially expressed genes for each treatment group, genes annotated within the Hallmark Inflammatory Response pathway of the MSigDB resource were used to construct an inflammatory gene list.

Signature scores for the corresponding gene lists were calculated using the singscore function from the package singscore (5, Ref) with the centerScore parameter set to FALSE. Seventeen overlapping genes between the two signatures (*ADRM1, PDE4B, HIF1A, MYC, SLC7A1, ACVR1B, BTG2, CD82, CDKN1A, HBEGF, IL1A, LIF, MXD1, PTPRE, RGS16, SPHK1, TNFSF9*) were removed from both lists. A linear model was fitted to compute R^2^ and F-test-derived *p*-values. Plots were generated with the R package ggplot2^105^.

### Macrophage-mediated phagocytosis assay

CD14+ monocyte isolation and differentiation into macrophages: Human blood was obtained from anonymized healthy volunteers and did not require specific ethics approval per University of Washington Human Subjects Division agreement involving Bloodworks Northwest, Seattle, USA. Following Ficoll density gradient centrifugation (300–500 g for 30 min, 20–30^o^C) on Day 0, peripheral blood mononuclear cells (PBMCs) were isolated from the buffy coat. Various cell fractions in the buffy coat were resuspended in lymphocyte separation medium (Corning #25072CV) and centrifuged at 400 g for 60 min. Enriched PBMCs at the interface were collected, washed with 1X PBS, counted, and peripheral blood monocytes were isolated using CD14+ beads and LS columns (Miltenyi Biotec, #130-050-201) per manufacturer’s recommendation. For monocyte maturation, purified CD14+ monocytes were cultured and allowed to adhere in non-treated 10 cm tissue culture plates at 1 ×10^6^ per ml in each well. Seeded cells were differentiated into monocyte-derived macrophages (MDMs; CD68 + CD80 + CD14-) by stimulating with grown media (Gibco AIM-V Medium #12055091) supplemented with macrophage colony-stimulating factor (M-CSF at 100 ng/ml; R&D 215-GM-010) for 7 days.

Apoptosis induction: On Day 4, ATRN-119 (100 nM) was added to WaGa or MKL2 cells (3–5 million cells per 6 cm dish, in triplicate). MCC13-HL or MCC26 cells (1.5 million per dish) were treated with 250 nM of ATRN-119. After 45–60 min, cells received 4 Gy of ionizing radiation exposure and incubated for 72 h in a humidified 5% CO_2_ atmosphere until further use.

pHrodo labeling of tumor cells: To quantify phagocytosis, a fraction of target MCC cells (1 × 10^6^ per ml) harvested after 3 days of ATRi ± 4 Gy RT treatment was starved in RPMI with 2% FBS for 30 min on Day 7. Along with untreated controls, dead and live cell populations in each treatment plate were stained with pHrodo^TM^ Red (Invitrogen #P36600), a pH-sensitive, amine-reactive fluorophore, at a desired concentration of 20 ng/ml (stock prepared as 1000X in DMSO). After 30 min at room temperature, cells were washed thrice with ice-cold 1X PBS and resuspended at 1 million per ml in RPMI with 2% FBS.

For the adherent cell lines, trypsin (0.25%) was utilized for cell detachment prior to the washing step. Untreated or vehicle-treated cells tagged with pHrodo served as a control. The remaining treated cells were analyzed via flow cytometry for cell surface calreticulin performed with anti-human calreticulin conjugated to Alexa Fluor 647 fluorochrome (R&D IC38981R).

In vitro phagocytosis assay and FACS analysis: On Day 6, MDMs (2.0 × 10⁵ per well) were plated in non-treated 24-well plates using AIM-V medium supplemented with M-CSF (10 ng/ml) 24 h prior to co-incubation for engulfment of treated MCC cells. Harvested pHrodo-labeled target cell single cell suspensions were prepared at a minimum concentration of 1 × 10^6^ cells in 200 μl per well (RPMI with 2% FBS containing 0.2% penicillin-streptomycin), added to the wells on Day 7 and co-cultured for 1 h at 37^o^C (macrophage media was completely removed prior to this step). After incubation, all wells of the 24-well plates were washed twice with PBS, cells detached using trypsin, transferred to FACS tubes, and then stained with a CD68 (1:50; BioLegend #137013) and CD80 (1:100; Novus Biologicals #NBP1-43384APCCY7) antibody cocktail for 30 min at 4^o^C (PBS with 2% FBS and 1 mM EDTA buffer). Measurement of phagocytic receptor CD91 (1:50 dilution; Miltenyi Biotec, #130-111-413) fluorescence on stimulated macrophages was performed separately to validate calreticulin recognition (Supplementary Fig. 7). Acquisition of each antibody staining and pH-mediated change in pHrodo fluorescent intensity was performed with BD FACSCanto Ruo II flow cytometer after resuspension in PBS with 2% FBS and 1 mM EDTA. MDM identification was performed as previously reported^107^.

Data processing and visualization: Acquired .fcs files were manually gated for relevant populations using the FlowJo software. Unstained and stained macrophages for CD68 (PE), CD80 (APC-Cy7) and CD91 (APC) surface markers were used for MDM identification. For determination of target cell internalization, 10,000 MDM events presenting enhanced pHrodo fluorescent signal uptake were calculated per sample. A representative gating strategy is included in Supplementary Fig. 8. Phagocytosis was quantified as the percentage of MDMs that had engulfed at least one pHrodo-positive (red) MCC tumor cell. Analyzed data was exported for further analysis of each treatment group and then plotted to depict differential treatment effect.

### Statistical analyses

Unless otherwise stated, a two-sided *p*-value of 0.05 was considered the threshold for statistical significance following treatment induction. Across figures, significance is depicted as follows: *p* < 0.05 as (*), *p* < 0.001 (**). GraphPad Prism was used for data visualization and statistical testing (Mann-Whitney, unpaired or two-tailed t-tests), as specified in individual figure captions. Whole-transcriptome analyses were performed using R-based pipelines and corresponding package-specific statistical guidelines.

## Supplementary information

ATRi MCC_Bhakuni Nghiem npj Precision Oncology 8212026 742026 472026_Supplementary Info_Clean Version.

## Data Availability

The transcriptomic datasets generated and analyzed in this study are available in the Gene Expression Omnibus (GEO) under accession number GSE317774.
