# Supplementary material for: ATR inhibition induces NF-κB-dependent immunogenicity in Merkel cell carcinoma independent of cGAS-STING
Source: NPJ Precis Oncol. 2026 Sep 12;10:368. doi: 10.1038/s41698-026-01688-4 (PMC13638979; doi:10.1038/s41698-026-01688-4)
Supplement: Supplementary file 1 [file 41698_2026_1688_MOESM1_ESM.pdf]

ATR inhibition induces NF-κB-dependent immunogenicity in Merkel cell carcinoma independent of cGAS-STING

Rashmi Bhakuni[1], Jung Hyun Lee[2], Peter Goff[3], Daniel Jones[4], Long Nguyen[4,9], Rongxiang Lin[1,10], Evan W. Newell[4], Khalid A. Garman[5], Evan T. Hall[6], Oren Gilad[7], Isaac Brownell[5], Eric J. Brown[8], Paul Nghiem[1,4,6]

Institutions:
[1]Department of Dermatology, University of Washington, Seattle, WA, USA
[2]University of Washington, Seattle, WA, USA
[3]Department of Radiation Oncology, University of Washington, Seattle, WA, USA
[4]Fred Hutchinson Cancer Center, Vaccine and Infectious Disease Division, Seattle, WA, USA
[5]Dermatology Branch, National Institute of Arthritis and Musculoskeletal and Skin Diseases, National Institutes of Health, Bethesda, MD, USA
[6]Fred Hutchinson Cancer Center, Clinical Research Division, Seattle, WA, USA
[7]Aprea Therapeutics, Philadelphia, PA, USA
[8]University of Pennsylvania, Philadelphia, PA, USA

Current Affiliation:
[9]University of Michigan, Ann Arbor, MI, USA
[10]Pacific Northwest University of Health Sciences, Yakima, WA, USA

Corresponding author: Paul Nghiem, MD, PhD (pnghiem@uw.edu)

Supplementary

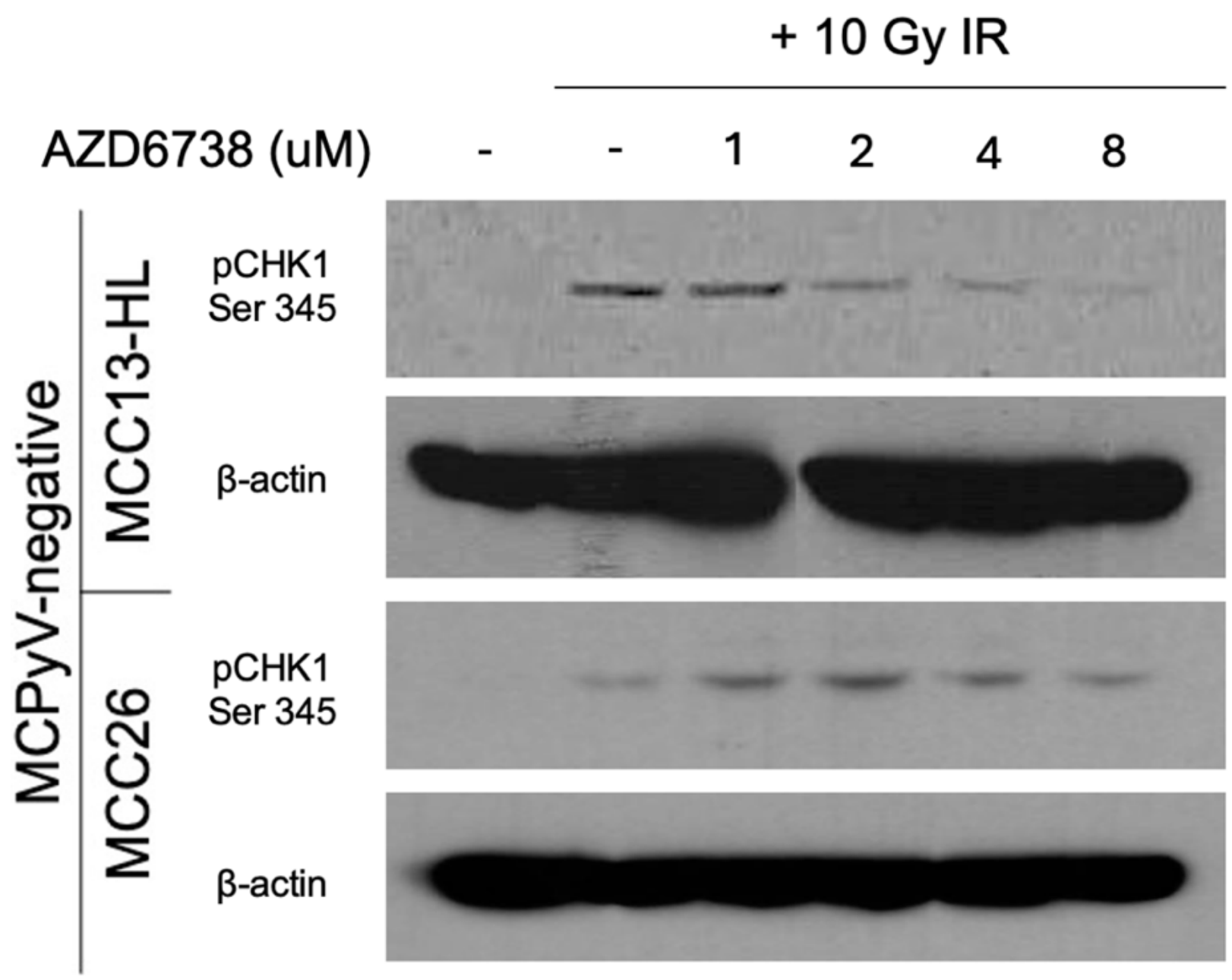

Supplementary Figure 1: Immunoblot analysis of higher-dose (>1 µM) AZD6738-induced inhibition of p-CHK1 Ser 345 in MCC13-HL and MCC26 cell lines. This figure supplements data shown in Fig 1d, corresponding to doses >1 µM. A decrease in p-CHK1 signal was observed at concentrations above 1 µM, with notable suppression at 4 µM in MCC13-HL and ~8 µM in MCC26. β-actin was derived from independent gels; MCC13-HL is depicted as a merged blot (two gels, due to space constraints), whereas MCC26 is shown as a single uninterrupted gel.

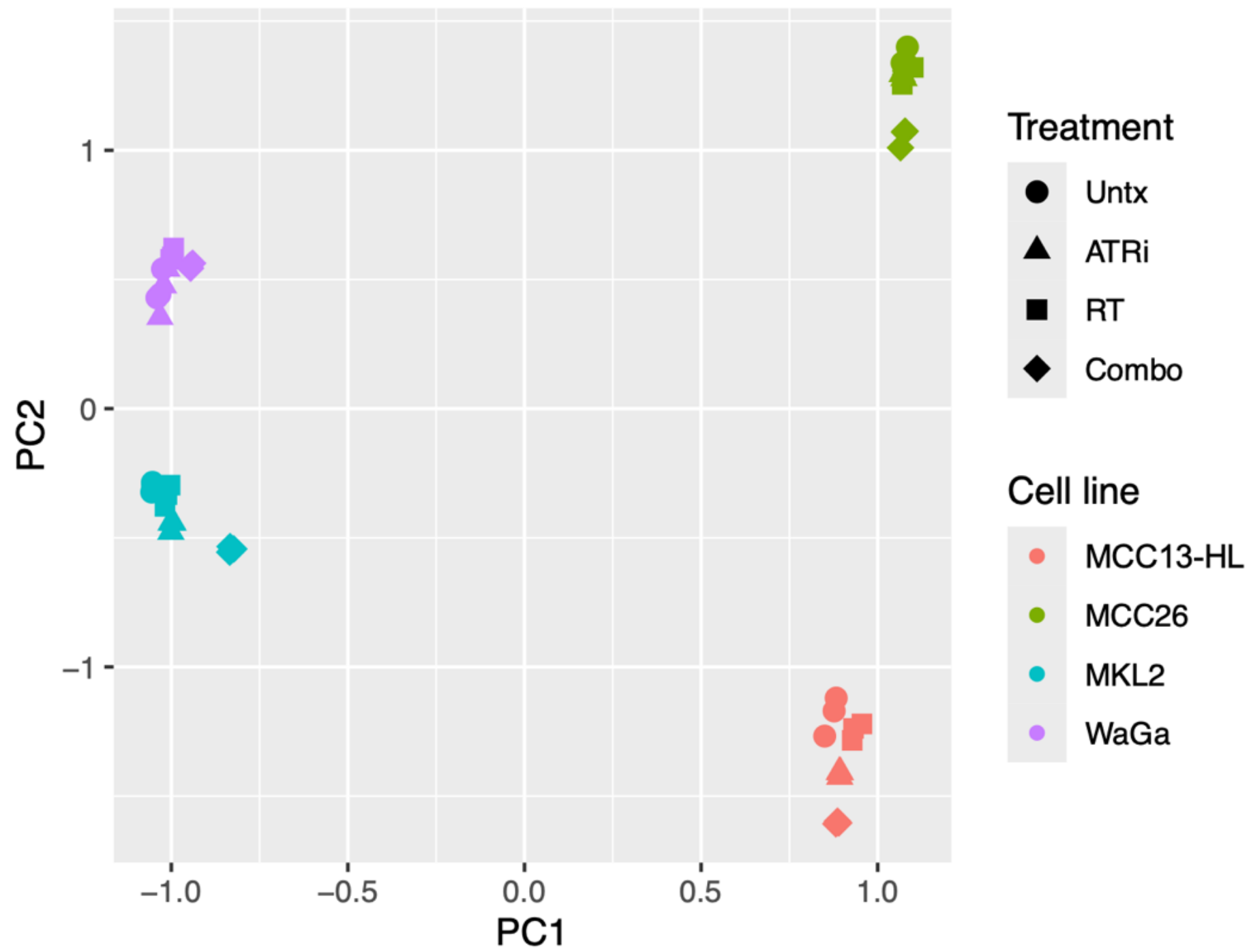

Supplementary Figure 2: Systematic evaluation of bulk-RNA sequencing data of MCC cell lines. PCA plot of normalized transcriptome data illustrates the variance among biological samples across different cell lines. Each color denotes a distinct cell line, with 12 samples per line representing four treatment conditions performed in triplicate. Samples cluster distinctly based on etiology indicating transcriptional differences consistent with viral status. Treatment groups are denoted by symbol: Untreated (circles), ATRi (triangles), RT (squares), and ATRi + RT (diamonds). Cell lines are denoted by color: MCC13-HL (pink), MCC26 (green), MKL2 (cyan), and WaGa (purple).

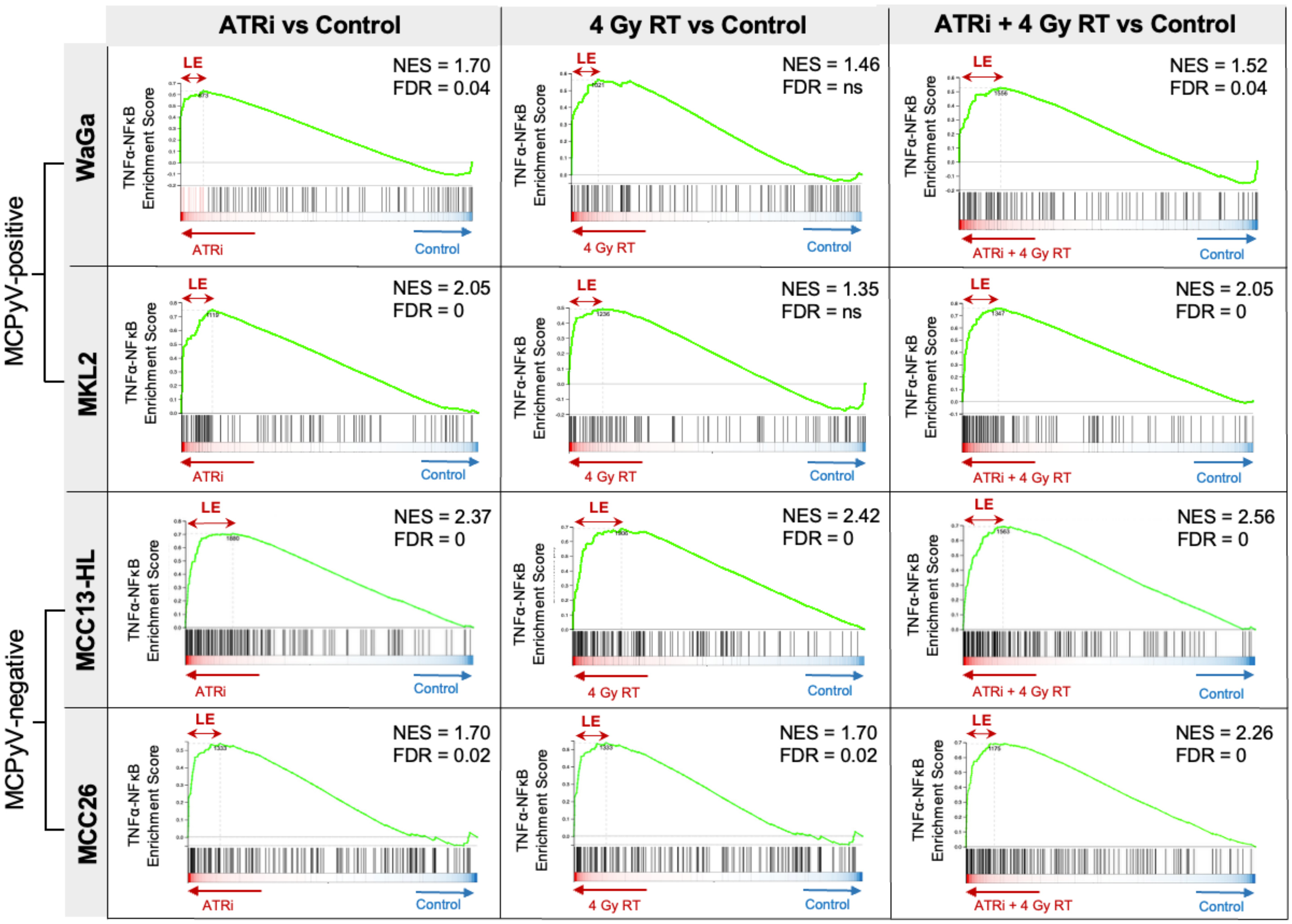

Supplementary Figure 3: ATR inhibition induces enrichment of the MSigDB hallmark TNF-NF-κB transcriptomic signature in MCC. GSEA results for the TNF-NF-κB hallmark pathway are shown for each treatment group versus control in representative MCPyV-positive and –negative cell lines. Normalized enrichment scores (NES) and false discovery rates (FDR) are indicated. The leading edge (LE) area represents high scoring gene subsets that contribute most to the enrichment signal and are tightly associated with corresponding treatment phenotype, as identified via GSEA analysis of the bulk RNA-seq dataset.

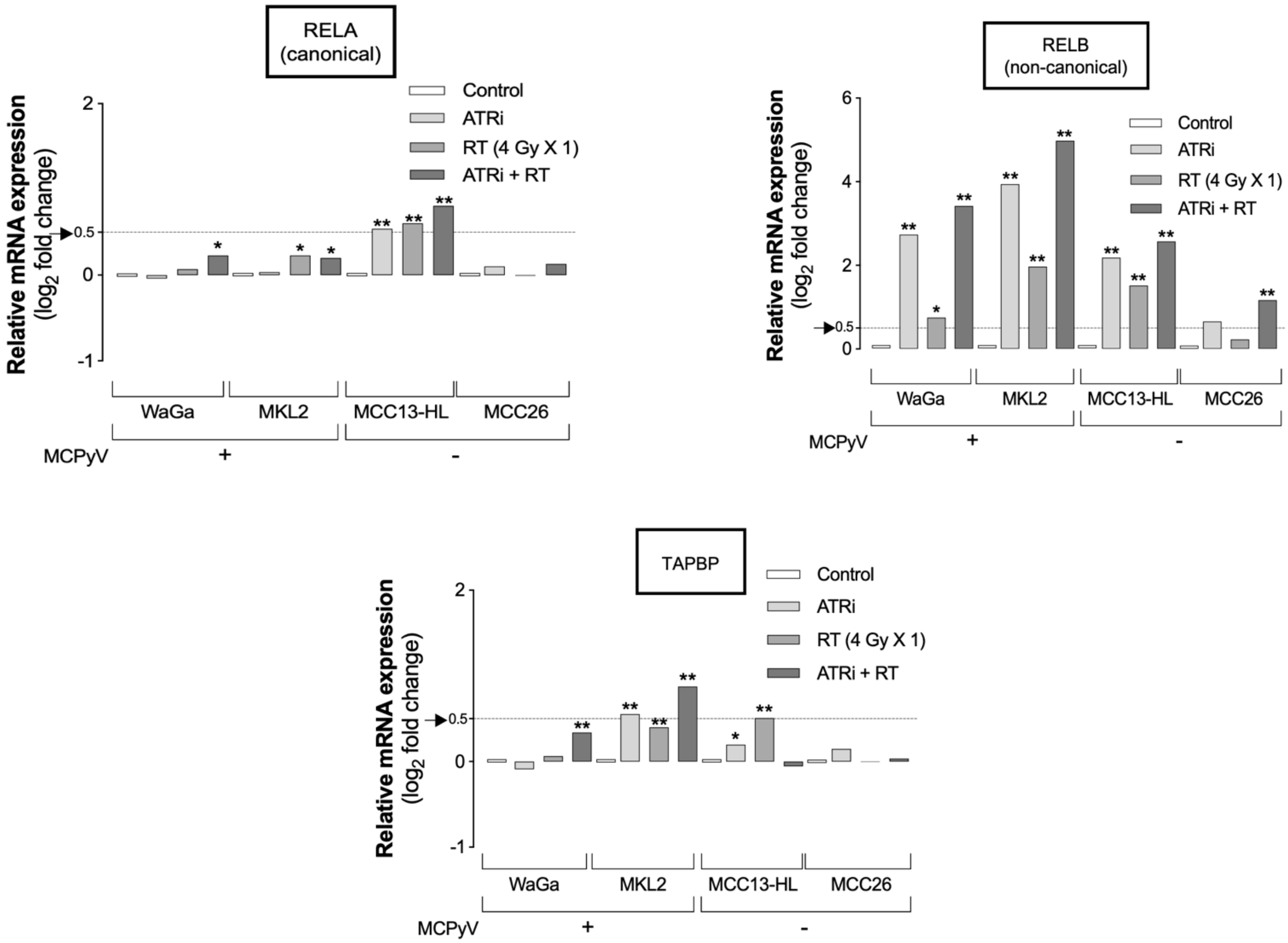

Supplementary Figure 4: Transcriptomic changes induced by ATR inhibition alone or in combination with radiation. In addition to Figure 2, these bar plots quantify differential expression of additional key genes involved in canonical and non-canonical NF-κB signaling pathways, as well as antigen presentation machinery ($log_2FC > 0.5$; $Q \leq 0.05$; *<0.05, **<0.001). Data are derived from bulk-RNA-sequencing of representative MCC cell lines treated with an ATR inhibitor, radiation, or the combination of both. Graphical coding: ATRi + RT, dark gray; RT alone, light gray; ATRi alone, opaque; Control, transparent.

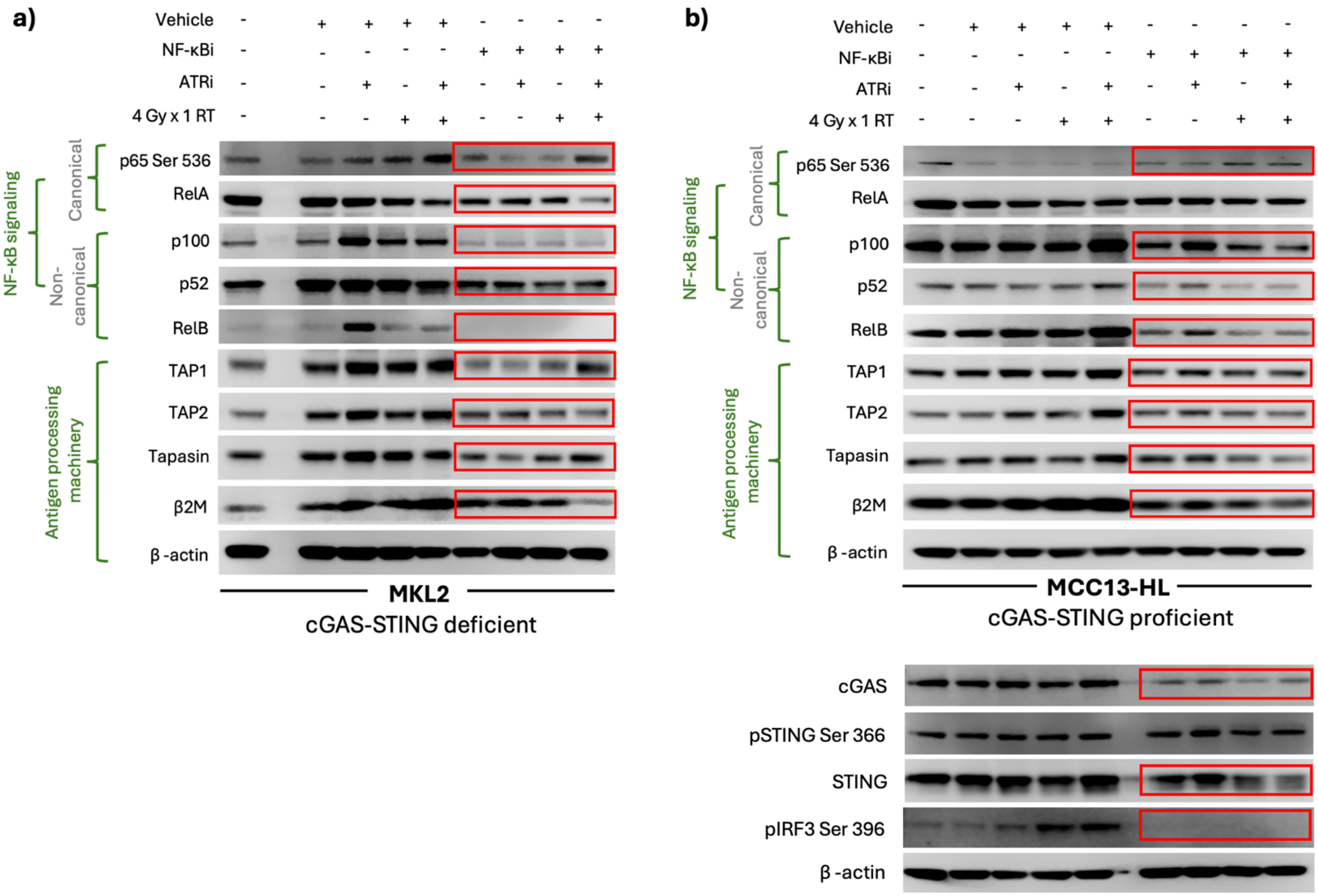

Supplementary Figure 5: ATR inhibition-induced antigen-processing and interferogenic nucleic acid-sensing pathways in MCC are NF-κB-dependent. a-b) Representative MCPyV-positive (MKL2) and MCPyV-negative (MCC13-HL) MCC cells were pre-treated for 24h with the NF-κB transcriptional activation inhibitor JSH-23 (25µM, prepared in DMSO), followed by ATRN-119 ± 10 Gy ionizing radiation as described earlier. Immunoblot analysis of harvested cells at 72 hours demonstrated that NF-κB blockade reversed the induction of key pathway components. Interestingly, this inhibition was accompanied by reduced stimulation of class I antigen-processing machinery with ATRi ± RT in both tumor cell lines. **b)** In nucleic-acid-sensing-proficient MCC13-HL cells, NF-κB blockade additionally diminished cGAS-STING activation, concordant with a loss of downstream IRF3 (Interferon Regulatory Factor 3) phosphorylation at Ser 396 residue, supporting an NF-κB-dependent mechanism for ATRi-mediated innate immune signaling.

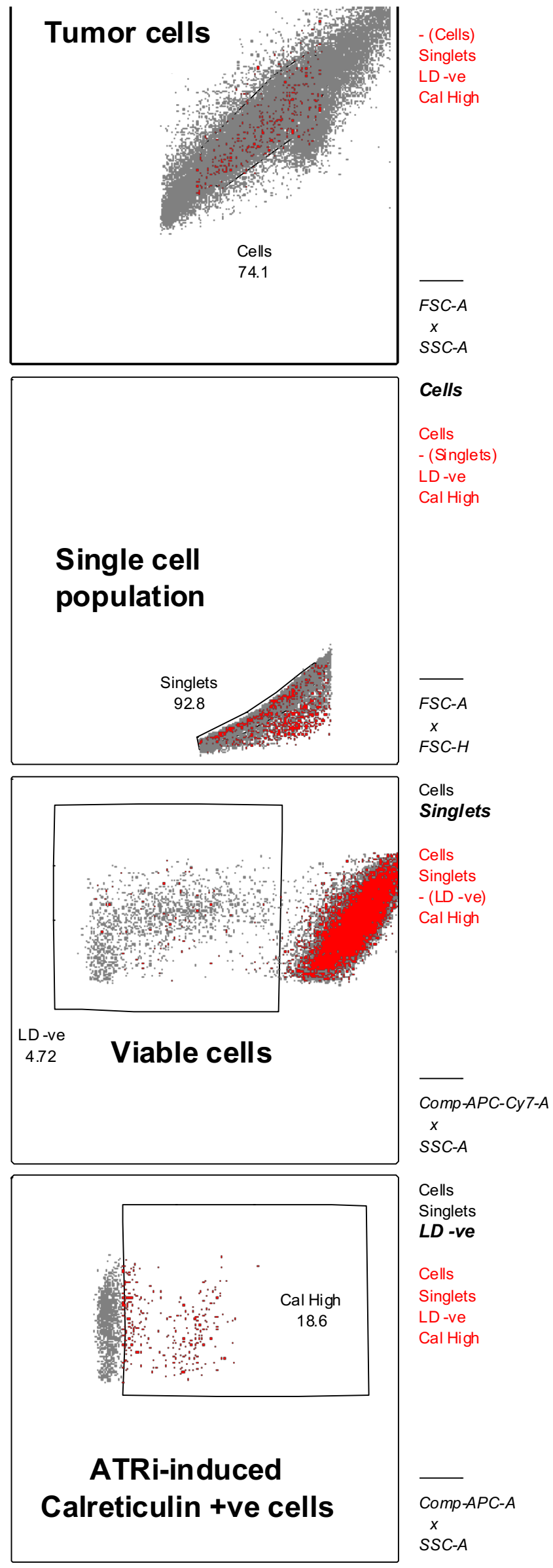

Supplementary Figure 6: ATR inhibitor treatment promotes tumor immunogenicity via upregulation of calreticulin expression on MCC tumor. MCC cells treated with ATRN-119 over multiple cell cycles were stained with a viability dye and an APC-conjugated anti-calreticulin antibody for flow cytometric analysis. The gating strategy used to detect calreticulin-positive cells at Day 12 post-treatment is depicted. Data were analyzed using FlowJo software.

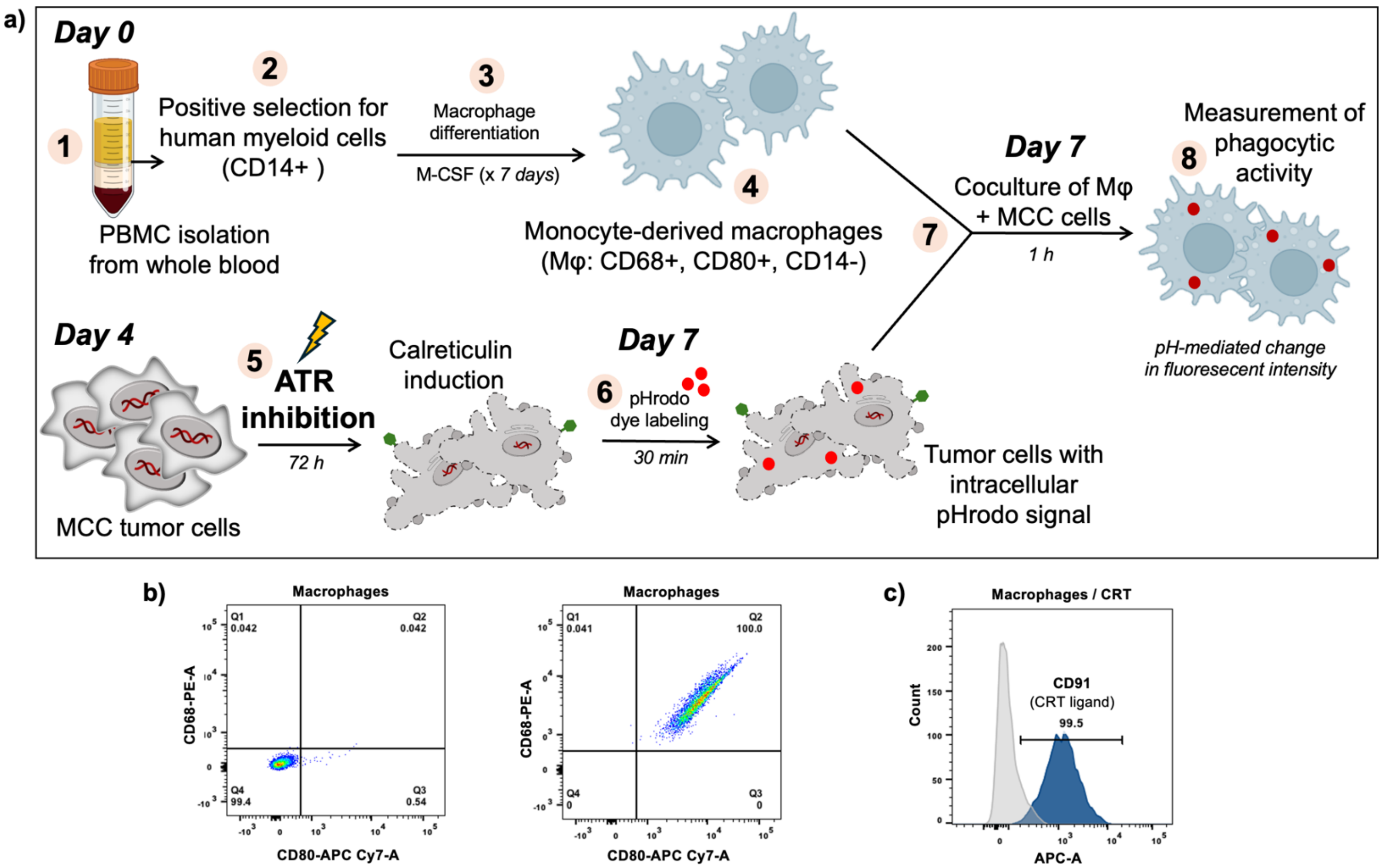

Supplementary Figure 7: ATRi ± low-dose radiation enhances phagocytosis of MCC tumor cells by human macrophages. a) Steps 1-8 summarize the experimental workflow for the generation of monocyte-derived macrophages (Mφ; Steps 1-4, Days 0-6) and their subsequent incubation with ATRi ± radiation-treated MCC cells to evaluate phagocytosis. On Day 4, MCC tumor cells were treated with an ATRi (100 nM for MCPyV-positive and 250 nM for MCPyV-negative) ± 4 Gy radiation (Step 5). 72h later, a subset of treated tumor cells were analyzed for calreticulin induction or labeled with pHrodo dye (Step 6). Labeled MCC cells were then co-cultured with activated human macrophages for 1 hour (Step 7). The percentage of macrophages that had engulfed at least one MCC tumor cell is shown (Figure 4b). Panels **b)** and **c)** represent fluorescence intensity changes in CD80, CD86 and CD91 surface markers measured on prepared macrophages.

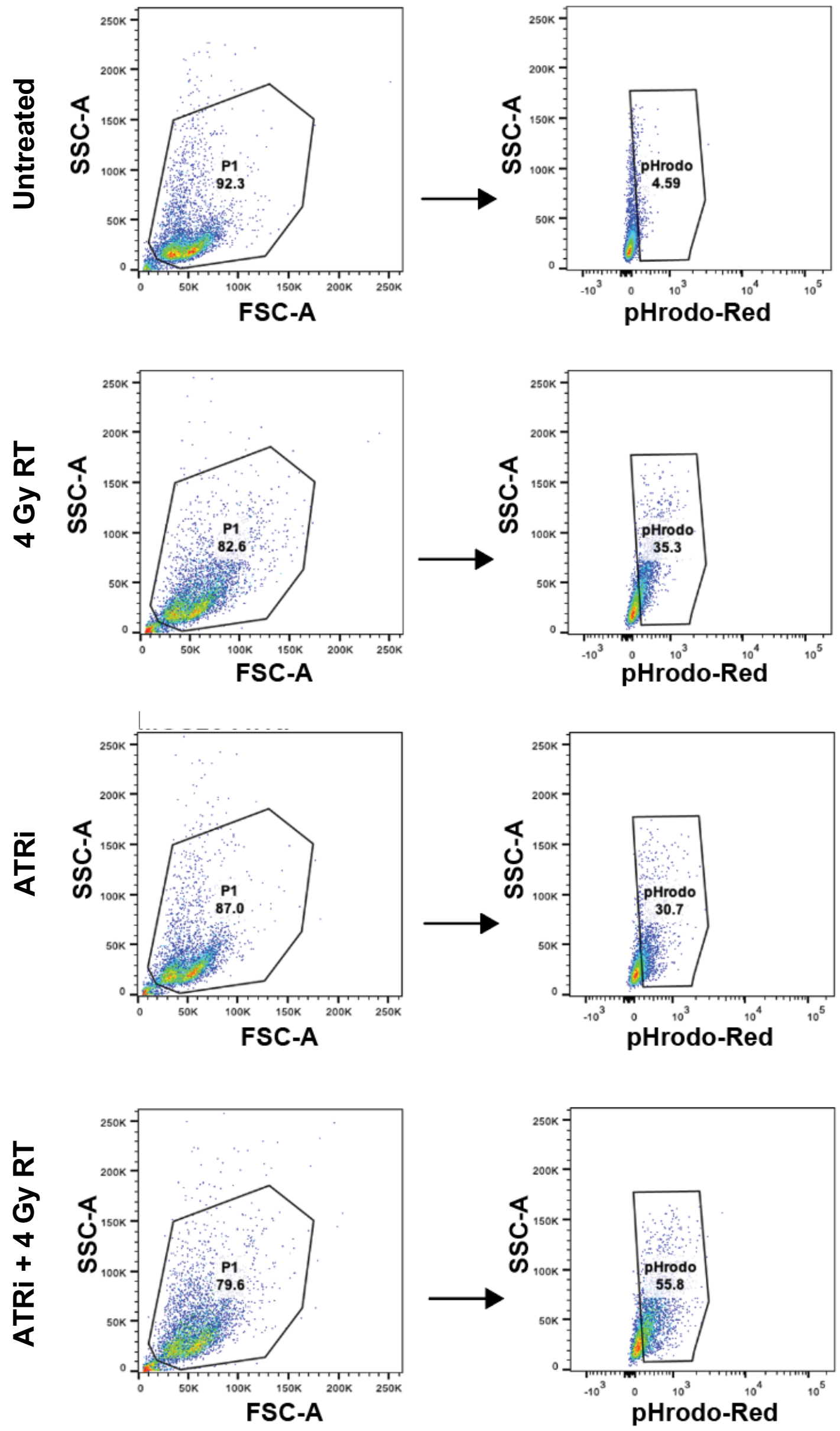

Supplementary Figure 8: ATR inhibition sensitizes irradiated MCC tumor cells to engulfment by macrophages. Representative gating strategies are shown for a phagocytosis assay performed using MCPyV-positive and –negative tumor cells treated with an ATR inhibitor, radiation, or the combination. 72h post-treatment, tumor cells were labeled with pHrodo dye and co-cultured with human monocyte-derived macrophages (as described in Supplementary Figure 7). The pH-sensitive increase in fluorescence intensity upon engulfment was quantified across each treatment condition. Across all tested cell lines, combined ATR inhibition and radiation significantly stimulated the percentage of macrophages that engulfed tumor cells.

a)

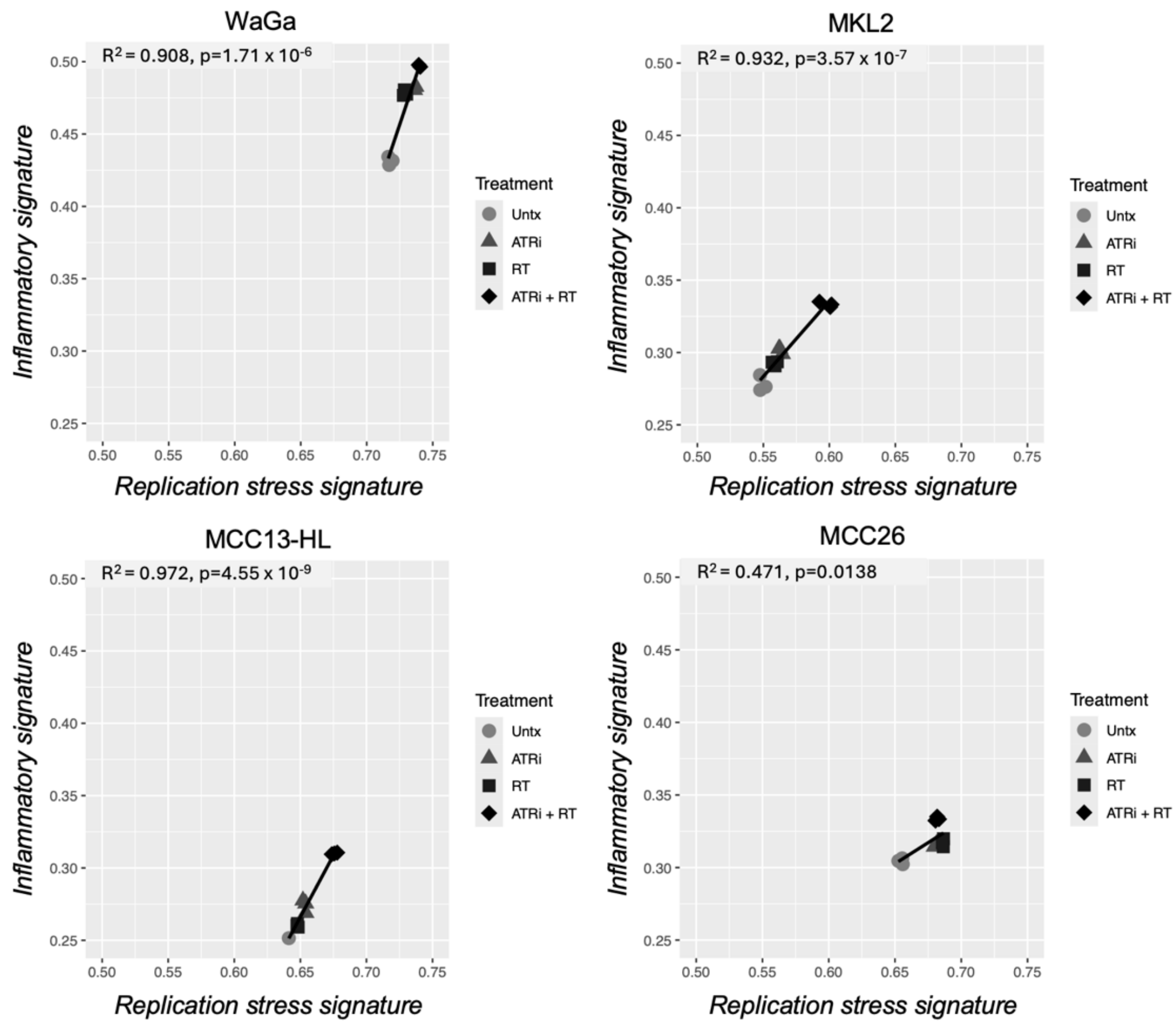

b)

WaGa RepStress Response			WaGa Inflammatory Response		
ATRi vs Control	RT vs Control	ATRi + RT vs Control	ATRi vs Control	RT vs Control	ATRi + RT vs Control
IER3	SMAD3	HMOX1	CCL2	IL4R	NFKB1
ZFP36L1	ZFP36L1	GADD45A	TNFRSF9	RNF144B	CCL2
SMAD3	DRAM1	ZFP36L1	RNF144B	TNFSF15	SERPINE1
GADD45A	GADD45A	ATF3	ITGB3	IRF1	IRF1
DRAM1	DDB2	PLK2	RIPK2	ITGB3	CXCL8
H1-2	PLK2	DRAM1		SERPINE1	LAMP3
ZMAT3	EPHA2	ZMAT3		ADM	BEST1
SESN1	ZMAT3	SMAD3		GPC3	ITGB3
DDB2	CCNK	CCNK		LAMP3	HRH1

MKL2 RepStress Response			MKL2 Inflammatory Response		
ATRi vs Control	RT vs Control	ATRi + RT vs Control	ATRi vs Control	RT vs Control	ATRi + RT vs Control
H1-2	SMAD3	HMOX1	CCL2	CCL2	BST2
SMAD3	IFI30	PPP1R15A	NFKBIA	BST2	IFITM1
IFI30	ADCY6	H1-2	NFKB1	TNFRSF1B	CCL2
IER3	TXNIP	IFI30	BST2	ITGB3	ADM
AURKA	H1-2	CEBPA	ICOSLG	ADM	IL1R1
HEXIM1	AURKA	OSGIN1	IL1R1	CMKLR1	NFKB1
TP63	FOS	IER3	TAPBP	NFKB1	CCL5
PPP1R15A	ZFP36L1	TLE3	ADM	CCL5	MMP14
BCL3	IER3	SMAD3	CXCL10	PTGER4	CXCL10

MCC13-HL RepStress Response			MCC13-HL Inflammatory Response		
ATRi vs Control	RT vs Control	ATRi + RT vs Control	ATRi vs Control	RT vs Control	ATRi + RT vs Control
IFI30	IFI30	CMPK2	LAMP3	CCL5	CCRL2
TXNIP	PPP1R15A	TXNIP	CCRL2	RTP4	LAMP3
DDIT3	DDIT3	ATF3	RTP4	LAMP3	SLC31A2
PPP1R15A	STX3	IFI30	CXCL10	IRF1	RTP4
CMPK2	TXNIP	CASP1	ROS1	CXCL10	CXCL10
ATF3	ATF3	KLF4	IRF1	IFITM1	ADM
STX3	SLC3A2	RNF19B	IFITM1	LY6E	ROS1
NDRG1	NDRG1	PPP1R15A	SEMA4D	CXCL11	IRF1
H1-2	CMPK2	PML	TNFRSF9	NOD2	IL10RA

MCC26 RepStress Response			MCC26 Inflammatory Response		
ATRi vs Control	RT vs Control	ATRi + RT vs Control	ATRi vs Control	RT vs Control	ATRi + RT vs Control
H1-2		PPP1R15A	SELE	C3AR1	SELE
IFI30		H1-2	C3AR1	RASGRP1	C3AR1
VDR		STX3	KCNJ2		RASGRP1
KLF4		IFI30	CXCL10		CXCL10
		ATF3	SLAMF1		IL7R
		DDIT3	CD48		GCH1
		VDR	MARCO		CXCL8
		TP63	LTA		IL6
		TSPYL2			CCL20

Supplementary Figure 9: A uniform scale to depict the correlation between replication stress and tumor-intrinsic inflammation across MCPyV-positive and –negative tumor cells with ATR inhibition. a) PCA revealed distinct spatial clustering for each MCC cell line. These line diagrams illustrate the standardized distribution of transcriptional responses, modeled along the x- and y- axes, across treatment conditions and biological replicates. These visualizations demonstrate consistent patterns of replication stress and inflammatory signaling in response to ATR inhibition, alone or in combination with radiation. Treatment groups are denoted by symbol: Untreated (circles), ATRi (triangles), RT (squares), and ATRi + RT (diamonds). **b)** Overview of gene composition in the curated RepStress and Inflammatory signature models. For each MCPyV-positive and –negative cell line, the tables display the top 10 statistically significant upregulated genes per signature across treatment conditions; light and dark green denote genes shared between conditions within WaGa and MKL2 or MCC13-HL and MCC26. Gene-level statistics were derived using the expression analysis package (see Methods) and included the moderated t-test statistic and the log-odds (B-statistic) of differential expression.

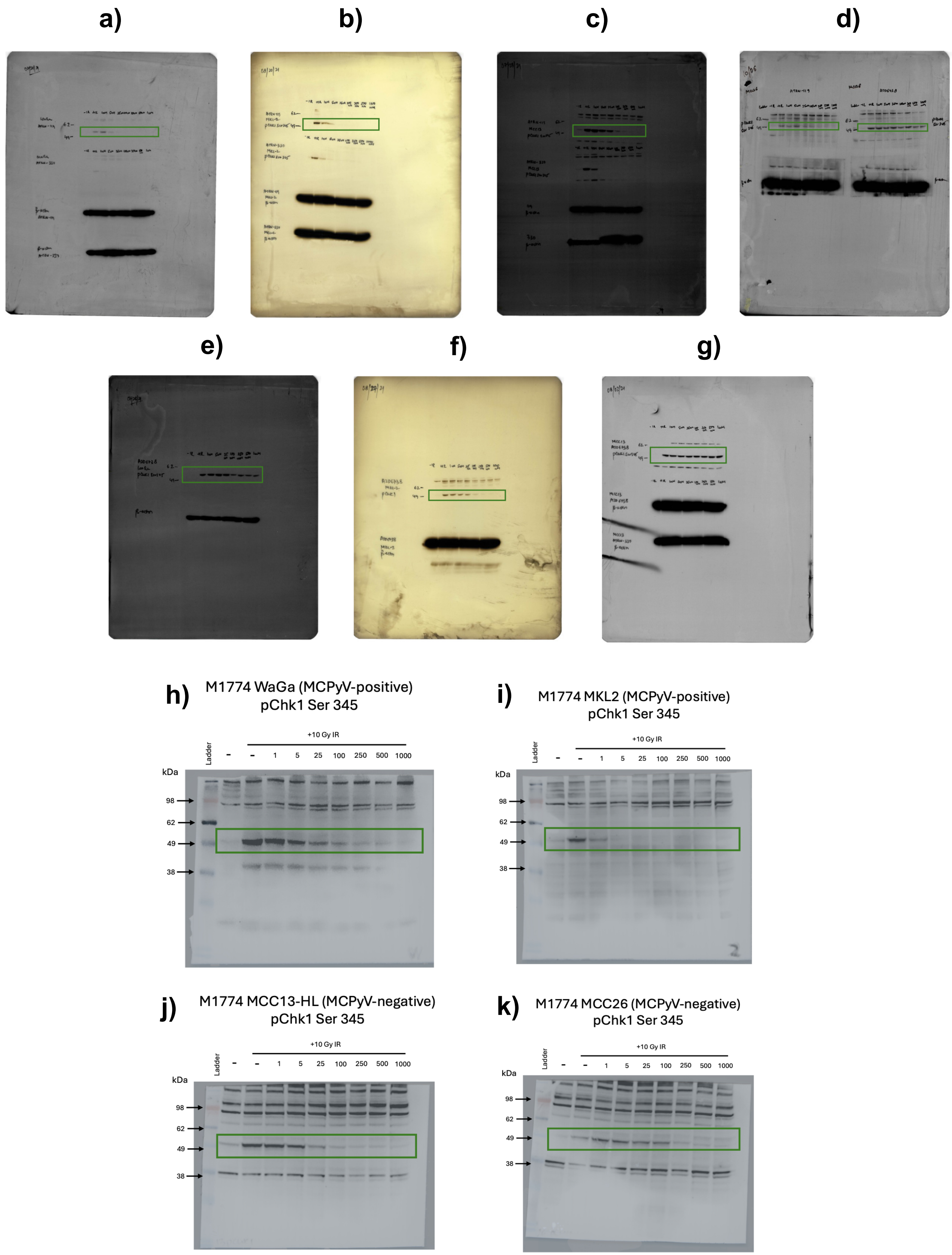
a)
b)
c)
d)
e)
f)
g)
h)
M1774 WaGa (MCPyV-positive)
pChk1 Ser 345
i)
M1774 MKL2 (MCPyV-positive)
pChk1 Ser 345
j)
M1774 MCC13-HL (MCPyV-negative)
pChk1 Ser 345
k)
M1774 MCC26 (MCPyV-negative)
pChk1 Ser 345
+10 Gy IR
Ladder
- - 1 5 25 100 250 500 1000
kDa
98
62
49
38

Supplementary Figure 10. Unprocessed immunoblot images (a-k) corresponding to the individual pCHK1 (Ser 345) lanes shown in Figure 1d. Whole-cell protein lysates from clinically relevant ATR inhibitor (ATRi)-treated, with or without 10 Gy ionizing radiation (IR), MCPyV-positive (WaGa and MKL2) and MCPyV-negative (MCC13-HL and MCC26) MCC cell lines were normalized, harvested, and electrophoresed under denaturing conditions. Detailed experimental procedures are provided in the Methods. Following incubation with the pCHK1 Ser 345 (labeled as pChk1 Ser 345) antibody, each PVDF membrane was visualized using either X-ray film or a digital imaging system. As shown, all experimental conditions included a negative control (lane 2) and a positive control (lane 3). Dose-dependent analyses for the ATR inhibitors ATRN-119, M1774, and AZD6738 are highlighted within transparent, green-bordered rectangles. The molecular weight protein standard is shown in lane 1.

MCC cell line(s)	MCPyV status	cGAS / STING status	Interferon response	Reference studies
WaGa	Positive	Deficient	Absent	• Pulliam et al. (JITC, PMID 39401968)<br>• Bhakuni et al. (Unpublished data)
MKL2	Positive	Deficient	Absent	• Liu et al. (PNAS, PMID 32482869)<br>• Pulliam et al. (JITC, PMID 39401968)
MCC13-HL	Negative	Proficient	Induced	• Liu et al. (PNAS, PMID 32482869)<br>• Bhakuni et al. (Supplementary Figure 5; Unpublished data)
MCC26	Negative	Proficient	-	• Liu et al. (PNAS, PMID 32482869)
cGAS-STING MCC functional studies described in Pulliam et al., and Bhakuni, et al. were carried out under the same laboratory conditions; led by Paul Nghiem, MD, PhD at University of Washington.				

Supplementary Table 1: Summary of cell lines and data supporting their cGAS-STING functional status. A tabular representation of key published and unpublished reference studies outlining tumor-intrinsic STING signaling status and its effect on downstream pathways.
